# A missense mutation in *Pitx2* leads to early-onset glaucoma via NRF2-YAP1 axis

**DOI:** 10.1038/s41419-021-04331-1

**Published:** 2021-10-29

**Authors:** Yeming Yang, Xiao Li, Jieping Wang, Junkai Tan, Bernie Fitzmaurice, Patsy M. Nishina, Kuanxiang Sun, Wanli Tian, Wenjing Liu, Xuyang Liu, Bo Chang, Xianjun Zhu

**Affiliations:** 1 Sichuan Provincial Key Laboratory for Human Disease Gene Study, Center for Medical Genetics, Sichuan Provincial People’s Hospital, University of Electronic Science and Technology of China, 610072 Chengdu, Sichuan China; 2 Henan Eye Institute, Henan Eye Hospital, People’s Hospital of Zhengzhou University, Henan Provincial People’s Hospital, 450003 Zhengzhou, Henan China; 3 Research Unit for Blindness Prevention of Chinese Academy of Medical Sciences (2019RU026), Sichuan Academy of Medical Sciences & Sichuan Provincial People’s Hospital, 610072 Chengdu, Sichuan China; 4 The Jackson Laboratory, Bar Harbor, ME 04609 USA; 5 Xiamen Eye Center, Xiamen University, 361006 Xiamen, Fujian China; 6 Department of Ophthalmology, Shenzhen People’s Hospital, the 2nd Clinical Medical College, Jinan University, 518020 Shenzhen, China

**Keywords:** Disease model, Neurodegeneration, Neurodegeneration

## Abstract

Glaucoma is a leading cause of blindness, affecting 70 million people worldwide. Owing to the similarity in anatomy and physiology between human and mouse eyes and the ability to genetically manipulate mice, mouse models are an invaluable resource for studying mechanisms underlying disease phenotypes and for developing therapeutic strategies. Here, we report the discovery of a new mouse model of early-onset glaucoma that bears a transversion substitution c. G344T, which results in a missense mutation, p. R115L in PITX2. The mutation causes an elevation in intraocular pressure (IOP) and progressive death of retinal ganglion cells (RGC). These ocular phenotypes recapitulate features of pathologies observed in human glaucoma. Increased oxidative stress was evident in the inner retina. We demonstrate that the mutant PITX2 protein was not capable of binding to Nuclear factor-like 2 (NRF2), which regulates *Pitx2* expression and nuclear localization, and to YAP1, which is necessary for co-initiation of transcription of downstream targets. PITX2-mediated transcription of several antioxidant genes were also impaired. Treatment with N-Acetyl-L-cysteine exerted a profound neuroprotective effect on glaucoma-associated neuropathies, presumably through inhibition of oxidative stress. Our study demonstrates that a disruption of PITX2 leads to glaucoma optic pathogenesis and provides a novel early-onset glaucoma model that will enable elucidation of mechanisms underlying the disease as well as to serve as a resource to test new therapeutic strategies.

## Introduction

Glaucoma, affecting 70 million people worldwide, is a group of neurodegenerative diseases characterized by the progressive death of retinal ganglion cells (RGCs) and atrophic excavation of the optic nerve, ultimately resulting in irreversible loss of vision [1, 2]. Elevated intraocular pressure (IOP) is thought to be a major risk factor for glaucoma, and patients with high-pressure glaucoma generally present defects in outflow of aqueous humor [3, 4]. IOP is maintained through a balance between aqueous humor secretion, by the ciliary body (CB), and drainage through the trabecular meshwork (TM), a porous tissue located in the iridocorneal angle [5]. An increased resistance to aqueous humor drainage can arise from developmental malformations of ocular structures and ultimately lead to early-onset glaucoma.

Early-onset glaucoma cases generally have strong genetic contributions and many disease-causing mutations that lead to pathogenesis have been identified [6]. For example, mutations in *CYP1B1, LTBP2, MYOC, FOXC1*, *GPATCH3*, and *TEK* have been associated with Primary Congenital Glaucoma (PCG) [7]. Moreover, mutations in *PAX6, PITX2, PITX3, FOXC1, FOXE3, EYA1, LMX1B*, and *MAF*, have been detected in patients with early-onset secondary glaucoma [8–12]. It should also be noted that glaucoma is a complex, heterogeneous disease likely to be the consequence of the interaction of multiple genes. This underlying complexity may hinder efforts to identify glaucoma-associated genes (or related mutations) and to uncover their pathogenic mechanisms. Identification of monogenic murine glaucoma models, may assist in addressing both of these issues. To this end, the Eye Mutant Resource screening program at The Jackson Laboratory (JAX) identified a glaucoma model caused by a mutation in *Pitx2*.

In humans, mutations in Paired-Like Homeodomain Transcription Factor 2 (*PITX2*) has been associated with Axenfeld-Rieger syndrome (ARS) (OMIM: 180500). *PITX2*, which encodes a member of the bicoid-like class of homeodomain (HD) transcription factors, play key roles in embryonic development and tissue morphogenesis. To date, a range of *PITX2* mutations have been identified in ARS patients including missense, nonsense, splicing mutations, and copy number variations [13, 14]. The majority of ARS-causing mutations are missense mutations within the HD region, which may impair DNA binding and decreasing transactivation activity [15]. ARS in humans is characterized by both systemic and ocular anomalies, such as corneal opacity, iris hypoplasia, corectopia, and iridolenticular adhesions [12]. These developmental malformations of the anterior segment in ARS patients lead to severe forms of early-onset glaucoma in ~50% of affected individuals.

Previous studies have suggested that *Pitx2* deficiency in mice lead to an arrest in anterior segment development in structures derived from the periocular mesenchyme, including the cornea, iris and outflow tract, which likely results from abnormal differentiation and migration of neural crest cells during the formation of anterior ocular structures [16–20]. Moreover, mice heterozygous for a *Pitx2* null allele recapitulate the anterior segment dysplasia and developmental glaucoma observed in ARS patients [21]. However, the role of PITX2 in maintenance of normal optic function in adults and the precise mechanisms underlying glaucomatous pathologies when PITX2 is disrupted remain largely unknown.

In this study, we identified a novel missense mutation, c. G344T, p. R115L, in *Pitx2*. In the homozygous state, mice bearing this mutation presents with bulging and distended eyes, a striking feature of glaucoma. Moreover, increased oxidative stress was evident in mutant retinas, which further activated persistent glial activation in the inner retina and RGC apoptosis. Mechanistically, the missense mutation disrupts protein interaction of PITX2 with NRF2 (regulating *Pitx2* expression and nuclear localization) and YAP1 (co-initiating transcription of downstream targets), leading to impaired PITX2-mediated transcription of several antioxidant genes, which activates the antioxidant response after ocular injury. Additionally, treatment with N-Acetyl-L-cysteine exhibited profound neuroprotective effect. Overall, this work describes a novel early-onset glaucoma model and sheds light on the pathogenesis of glaucoma in ARS.

## Materials and methods

### Mouse models

All animal experiments were approved by the Institutional Animal Care and Use Committee of Sichuan Provincial People’s Hospital (Chengdu, Sichuan, China) and The Jackson laboratory (JAX, Bar Harbor, Maine, USA) and conducted in accordance with the ARVO Statement for the Use of Animals in Ophthalmic and Vision Research.

The original mutant was identified in a N-ethyl-N-nitrosourea (ENU) chemical mutagenesis screen in the Mouse Resource for Craniofacial Research (http://craniofacial.jax.org/). Briefly, C57BL/6J male mice, G0, were treated with ENU (weekly 80 mg/kg dosage for 3 weeks), and bred to normal C57BL/6J females after returning to fertility. G1 male offspring were backcrossed to unmutagenized C57BL/6J females and their G2 female offspring were backcrossed to their G1 sires to generate a population of G3 mice that were screened for visible phenotypes. A G3 mutant with a shortened face and enlarged eyes was identified. This mutant was backcrossed again to C57BL/6J and the short face phenotype segregated from the ocular phenotype. This mutant was named early-onset glaucoma (*egl1*) and was bred to homozygosity and subsequently maintained by sibling intercrosses (C57BL/6J-*Pitx2*^*egl1*^/Boc; stock No. 004240). The *egl1* mutant mice in this study were bred and maintained in standardized conditions of the Research Animal Facilities at The Jackson Laboratory (JAX). They were provided with a NIH31 6% fat chow diet and acidified water, in a pathogen-free vivarium environment with a 14-hour light/10-hour dark cycle.

### Clinical evaluation and electroretinography

Eyes of all mice used in the characterization studies and linkage crosses were dilated with 1% atropine ophthalmic drops (Bausch and Lomb Pharmaceuticals Inc., Tampa, FL, USA) and were evaluated by indirect ophthalmoscopy with a 78-diopter lens. Fundus photographs were taken with a Micron III in vivo bright field retinal imaging microscope equipped with image-guided OCT capabilities (Phoenix Laboratories, Inc, Phoenix, AZ, USA). The intraocular pressure (IOP) was measured by an induction–impact tonometer (TonoLab Colonial Medical Supply, Londonderry, NH, USA). IOP was measured immediately after induction with an intraperitoneal injection of xylazine (80 mg/kg) and ketamine (16 mg/kg) in normal saline (~5 min). The mouse was gently restrained by hand on an adjustable platform, and the eye was oriented to align the probe tip with the optical axis of the eye at about 2-mm distance under dissecting scope. All tested animals with the same age were randomly divided into the experimental groups. Five consecutive IOP readings were averaged.

For electroretinographic evaluation of mutants, following an overnight dark adaptation, mice were anesthetized with an intraperitoneal injection of xylazine (80 mg/kg) and ketamine (16 mg/kg) in normal saline. Additional anesthetic was given, if akinesia was inadequate. The equipment and protocol used here have been previously described [22]. Briefly, dark-adapted, rod-mediated ERGs were recorded with the responses to short-wavelength flashes over 4.0-log units to the maximum intensity by a photopic stimulator. Cone-mediated ERGs were recorded with white flashes after 10 min of complete light adaptation. The signals were sampled at 0.8 ms intervals and averaged.

### Whole-exome sequencing (WES)

DNA purification, library construction, deep Next Generation sequencing, and data quality control was performed by the Jackson Laboratory’s Genome Technologies service, and data analysis and annotation were performed by the Computational Sciences Biostatistics service. Purified genomic DNA from *egl1/egl1* mice and C57BL/6J controls were used to create libraries for whole-exome sequence (WES) capture. High-quality reads were mapped to the mouse genome (GRCm38, mm10) and the resulting alignment was sorted by coordinates and further converted to binary alignment map (BAM) format by Picard v1.95 SortSam utility (http://picard.sourceforge.net). Variants with total read depth >5X with the SNP quality score >50 were included and variants in non-coding regions and synonymous variants were excluded. The filtered candidate genes are shown in Table S1.

### Gene mapping, sequencing, and genotyping

To determine the chromosomal location of the *egl1* mutation, we mated *egl1/egl1* mice to DBA/2J mice. The resultant F1 mice, which did not exhibit retinal abnormalities, were backcrossed to B6-*egl1/egl1* mice to produce N2 mice. Tail DNA was isolated as previously reported [23]. A genome-wide scan of pooled DNA from 12 affected and 12 unaffected mice was carried out using 48 microsatellite markers [24]. The *egl1* phenotype cosegregated with markers on Chromosome 3. Subsequently, DNAs of 93 N2 offspring were genotyped using microsatellite markers to develop a fine structure map of the Chromosome 3 region. Microsatellite markers *D3Mit106 and D3Mit291* were used to genotype individual DNA samples. The causative mutation was identified by comparing the whole-exome sequences from a homozygous *egl1* mutant and control [25]. Between *D3Mit106* and *D3Mit291* on Chromosome 3, a unique point mutation c.G344T of *Pitx2* was identified in the filtered data of WES (Table S2), in *egl1/egl1* but not in wild-type DNA. Allele-specific PCR (AS-PCR) was used on genomic DNA to confirm the presence of the *Pitx2*^*egl1*^ mutation. Three oligo primers were selected from exon 2 of the *Pitx2* gene with [G/T] single base change (WT = G, *egl1* mutant = T) using a web-based software “Web-based Alelle-Specific Primer” (http://bioinfo.biotec.or.th/WASP): wild-type (W), mutant (M) and common (C) primers: W reverse primer: CGAGTGGACATGTCTGGGTAAC; M reverse primer: CGAGTGGACATGTCTGGGTAAA; C forward primer: AGCAAGGAAAGAATGAGGAT, and the PCR product size 145 bp (base pairs). The AS-PCR assay was conducted in two parallel experiments: (1) “mutant + common” primer experiment (MC) and (2) “wild + common” primer experiment (WC). The PCR assay for *Pitx2*^*egl1*^ mutation was performed in 10 µl reactions. The PCR conditions were as followed: initial denaturation for 3 min at 94 ^o^C followed by 36 cycles of denaturation for 15 s at 94 °C, annealing for 2 min at 55 °C, extension for 2 min at 72 °C, and a final extension step for 7 min at 72 °C. PCR products were run on 1.5% SeaKem agarose gel.

### Generation of knock-in mouse model by CRISPR/Cas9

The CRISPR/Cas9 oligonucleotide directed approach was used to generate the *Pitx2* p.R115L knock-in (KI) mice. The gRNA (CTTCGCGAGTGGACATGTCTGGG) designed to mouse *Pitx2* gene together with Cas9 mRNA and a donor oligo (CAGAGGACTCATTTCACTAGCCAGCAGCTGCAGGAGCTGGAAGCCACTTTCCAGAGAAACCTCTATCCAGACATGAGTACTCGCGAAGAAATCGCCGTGTGGACCAACCTTACCGAAGCCCGAGTCCGGGTAGGAGCCAGCACCCAATCTGGGAAAACGAGGGGGCCGAGGCC) containing the R115L (CGC to CTC) mutation were co-injected into C57BL/6J mouse zygotes to generate targeted knock-in offspring. F0 founder animals were mated to C57BL/6J wildtype mice and subject to sequence analysis. Positive F1 offspring (*Pitx2*^R115L^, strain name C57BL/6J-*Pitx2*^*em1ZXJ*^, hereafter named *Pitx2*^*KI*^) were backcrossed to C57BL/6J for four generations and then intercrossed to generate heterozygous and homozygous offspring. After PCR amplification of the targeted region, homozygotes (*Pitx2*^*KI*^), heterozygotes (*Pitx2*^*KI/+*^), and wild-type (*Pitx2*^*WT*^) could be determined by Sanger sequencing analysis.

### Genotyping of *Pitx2*^*KI*^ mice

Genomic DNA was isolated either from tail tips or from blood using the QIAamp DNA Blood kit (QIAGEN) according to the manufacturer’s instructions. WES results or genotypes were confirmed by Sanger sequencing with the following primers (Sangon Biotech, Shanghai, China): Pitx2-Forward primer (F1): 5’-CGGTAGAGAGGTTGTAGATGGGAGTCT-3’; Pitx2-Reverse primer (R1): 5’-GCAGAGAGCCGCTGAGGTTGTAG-3’. All PCR amplification was performed using a master mix (Invitrogen, USA). The first cycle consisted of 95 °C for 5 min, followed by 32 cycles of 95 °C for 15 s, 60 °C for 30 s and 72 °C for 30 s. The PCR products were purified by FastAP Thermosensitive Alkaline Phosphatase (Thermo Scientific Fermentas), and directly sequenced using BigDye version 3.1 and an ABI 3730 automated sequencer (Applied Biosystems) according to the manufacturer’s instructions.

### Plasmids and site-directed mutagenesis

The recombinant expression plasmids, pcDNA3.1-PITX2-Flag, pcDNA3.1- Nrf2-HA tag, and pcDNA3.1-YAP1-HA tag were purchased from Youbio Inc. (Youbio, Changsha, China). Point mutation c. G344T was introduced into the WT *Pitx2* cDNA by site-directed mutagenesis using a QuikChange Lightning Site-Directed Mutagenesis Kit (Agilent Technologies, Santa Clara, CA, USA) with a complementary pair of primers: F: 5’-CCACTTTCCAGAGAAACCTCTACCCAGACATG-3’ and R: 5’-CATGTCTGGGTAGAGGTTTCTCTGGAAAGTGG-3’. The recombinant plasmids containing PITX2 (R115L)-Flag fusion constructs were sequenced to confirm the desired mutation and to exclude any other sequence variations.

### Cell culture and transfection

HEK293T and COS-7 cells were purchased from National Infrastructure of cell line Resource (Wuhan, China) and were recently authenticated by STR profiling. They were cultured in DMEM with high glucose (Hyclone, South Logan, UT, USA) supplemented with 10% fetal bovine serum (Gibco, Grand Island, NY, USA) and 100 U/ml penicillin/streptomycin (Invitrogen, Waltham, MA, USA) in an incubator set to 37 °C with 5% CO_2_. For transfection, cells were seeded in plates (Corning, NY, USA) and transiently transfected with Flag-tagged PITX2^WT^/PITX2^R115L^ or HA-tagged NRF2/YAP1 plasmid using Lipofectamine 3000 (Invitrogen, CA, USA) according to the manufacturer’s instructions, and the cell lysis were harvested after 48 h.

### Immunocytochemistry

COS-7 cells were seeded in 24-well plates (Corning, Corning, NY, USA) and transfected at 70% confluency with constructed vectors or empty vectors using Lipofectamine 3000 (Invitrogen, CA, USA) according to the manufacturer’s instructions. Cells were harvested after 48 h and fixed in 4% paraformaldehyde for 15 min at room temperature. After blocking with 1× PBS containing 5% normal goat serum and 0.2% Triton X-100, cells were incubated with specific antibodies at 4 °C overnight. The primary antibodies used are shown in Table S3. AlexaFluor 594/488-conjugated goat anti-mouse/rabbit secondary antibody (Cat# A11005 and A11008, Invitrogen, Waltham, MA, USA, 1:500 dilution) was applied and nuclei were counter-stained with DAPI (Cat# D8417, Sigma, St Louis, MO, USA).

### Luciferase assay

The luciferase assays to detect the transcriptional activity of PITX2 were performed as described previously [26, 27]. The human LEF-1 promoters were constructed in the luciferase vector as previously described [28, 29]. SV-40 β-galactosidase reporter plasmid was used as a control for transfection efficiency. 293T cells were prepared and mixed with 2.5 µg of expression plasmids, 5 µg of reporter plasmid and 0.5 µg of SV-40 β-galactosidase plasmid in 60 mm culture dishes. After incubating for 48 h, transfected cells were lysed and assayed for reporter activities. Luciferase activity was measured using reagents from Promega (Cat# E1500, Madison, WI, USA). β-galactosidase was measured using Galacto-Plus reagents (Cat# T2118, Invitrogen, Waltham, MA, USA). All luciferase activities were normalized to β-galactosidase activity and are shown as mean-fold differences relative to empty luciferase plasmids.

### RNA-sequencing analysis

RNA-sequencing analysis was performed on four independent biological replicates from four wild-type (WT) and four *Pitx2* mutant retina at 2 months of age. After harvesting, both retinas for each animal were collected and immediately frozen. RNA was extracted using RNeasy Mini kits (QIAGEN). RNA integrity and concentration were evaluated using a BioAnalyzer 2100 with RNA 6000 Nano Kit (Agilent Technologies, Santa Clara, CA, USA). For library preparation, a total of 2 μg RNA per sample was used as input material for the RNA sample preparations. Sequencing libraries were generated using NEB Next, Ultra RNA Library Prep Kit for Illumina® (Cat# E7530L, NEB, USA), following the manufacturer’s recommendations, and index codes were added to attribute sequences to each sample. Briefly, mRNA was purified from total RNA using poly-T oligo-attached magnetic beads. Fragmentation was carried out using divalent cations under elevated temperature conditions in NEBNext First-Strand Synthesis Reaction Buffer (5×). First-strand cDNA was synthesized using random hexamer primers and RNase H. Second-strand cDNA synthesis was subsequently performed using dNTPs, DNA polymerase I and RNase H. The library fragments were purified with QiaQuick PCR kits and eluted with EB buffer, and then terminal repair, A-tailing and addition of an adapter were implemented. The RNA concentration of the library was measured using a Qubit RNA Assay Kit in Qubit 3.0 and the library was diluted to 1 ng/μl. Insert sizes were assessed using the Agilent Bioanalyzer 2100 system (Agilent Technologies), and the qualified insert size was accurately quantified using a StepOnePlus™ Real-Time PCR System (library valid concentration > 10 nM). The clustering of the index-coded samples was performed on a cBot cluster generation system using a HiSeq PE Cluster Kit v4-cBot-HS (Illumina, CA, USA) according to the manufacturer’s instructions. After cluster generation, the libraries were sequenced on an Illumina HiSeq 2500 platform, and 150 bp paired-end reads were generated. All gene expression values from RNA-seq were converted to a log2 value and analyzed further. Then, a P value of less than or equal to 0.05 was considered significant. The raw sequence data have been deposited in the Genome Sequence Archive (Genomics, Proteomics & Bioinformatics 2021) in National Genomics Data Center, Beijing Institute of Genomics, Chinese Academy of Sciences, under accession number CRA005112 that are publicly accessible at “https://ngdc.cncb.ac.cn/gsa”.

### RNA extraction and quantitative PCR

Optic nerve total RNA was extracted using TRIzol reagent (Sigma, Saint Louis, MO, USA) as recommended by the manufacturer. First-strand cDNA was synthesized using iScript cDNA Synthesis Kit (Bio-Rad, Hercules, California, USA). Quantitative PCR was carried out using iTaq SYBRMix (Bio-Rad, Hercules, California, USA) and a CFX384 Touch Real-Time PCR Detection System (Bio-Rad, Hercules, California, USA). Primers were designed using Primer3Plus or from published resources. Table S4 shows the specific primer sequences used. Four biological replicates were used for each sample. All target genes were normalized to actin mRNA levels and fold change were calculated by performing delta-delta Ct analysis [30].

### Histological analysis

For haematoxylin and eosin staining (H&E), enucleated eyes from control and mutant mice were fixed overnight in 1.22% glutaraldehyde and 0.8% paraformaldehyde in 0.08 M phosphate buffer, embedded in paraffin and then cut in 5 μm sections. To ensure sections used for quantification came from the same eccentricity, the globe was embedded in the same orientation. Sections that encompassed the optic nerve (ON) were selected for staining with haematoxylin and eosin according to standard protocol.

### Immunohistochemistry

For immunohistochemistry, eyes were removed from euthanized mice by intraperitoneal injection of pentobarbital (75 mg/kg), and by cervical dislocation and fixed in 4% paraformaldehyde in 100 mM phosphate buffer (pH 7.4) for 1 h at 4 °C, followed by cryoprotection in 30% sucrose for 2 h. Lens were removed and eyes were embedded in optimal cutting temperature solution (OCT) and sectioned at 10 μm thickness. After blocking and permeabilization with 10% normal donkey serum and 0.2% Triton X-100 in phosphate buffer for 1 h, the sections were labeled with the primary antibody at 4 °C overnight. The primary antibodies used are shown in Table S3. The sections were rinsed in PBS three times and Alexa Fluor 594/488-conjugated goat anti-mouse/rabbit secondary antibody (Cat# A11005 and A11008, Invitrogen, Waltham, MA, USA, 1:500 dilution) was applied and nuclei were counter-stained with DAPI (Cat# D8417, Sigma, St Louis, MO, USA) for 1 h at room temperature. Images were captured on a Zeiss LSM 800 confocal scanning microscope. In addition, the expression levels of CD68 and GFAP were compared between WT and mutant retinas by quantifying their fluorescence intensities using ImageJ software.

### Retinal flat mounts and RGC count

Retinas were dissected from enucleated eyes and flattened. Retinas were immersed in 4% PFA for 24 h at 4 °C, then blocked in PBS containing 1% bovine serum albumin and 0.5 % Triton X-100 and incubated with polyclonal rabbit anti-Brn3a antibody (Cat#ab245230, Abcam, Cambridge, USA, 1:200 dilution) for 12 h at 4 °C. After several washes, the retinas were incubated with the secondary antibody, AlexaFluor 488-conjugated goat anti-rabbit secondary antibody (Cat#A11008, Invitrogen, Waltham, MA, USA, 1:250 dilution) for 4 h at room temperature, and subsequently washed with PBS. To precisely quantitate the loss of RGCs, each retinal quadrant was divided into four zones—nasal, temporal, superior, and inferior—each located 1 mm from the optic nerve head. RGCs were counted in each unit area by two investigators in a blinded fashion, and the scores were averaged.

### Semithin-section processing of the optic nerve

Optic nerves were dissected from eyes and placed in a solution containing 4% paraformaldehyde and 0.2% glutaraldehyde for 24 h. After rinsing three times in 0.1 M phosphate buffer (pH 7.4) for 15 min each, tissues were fix with 1% osmic acid and 0.1 M phosphate buffer (pH 7.4) at room temperature (20 °C) for 2 h. Osmic acid fixed tissues were rinsed three times in 0.1 M phosphate buffer (pH 7.4) for 15 min each, and dehydrated in graded concentrations of ethanol. The dehydrated tissues were embedded in acetone: 812 embedding medium = 1:1 for 2–4 h, and in acetone: 812 embedding medium = 1:2 infiltration, overnight, and finally in 812 embedding medium for 5–8 h. Samples were inserted into an embedding plate containing 812 embedding medium, and placed overnight in an incubator at 37 °C, followed by polymerization at 60 °C for 72 h. Semithin sections (1 μm) were cut transversely from segments of optic nerves caudal to the eyeball and stained with 1% toluidine blue.

### TUNEL assay

Apoptotic cell death was detected in prepared frozen sections by the terminal deoxynucleotidyl transferase-mediated biotinylated UTP nick end labeling (TUNEL) assay according to the manufacturer’s protocol (Cat#11684795910, Roche Diagnostics, Indianapolis, IN). Images were captured on a Zeiss LSM 800 confocal scanning microscope.

### Immunoblotting

Samples for western analysis were lysed in standard RIPA lysis buffer (50 mM Tris-HCl, 150 mM NaCl, 1% Triton X-100, 0.5% sodium deoxycholate, 0.1% SDS, pH 7.4) supplemented with Complete Protease Inhibitor Cocktail (Roche). The protein content of the samples were quantified by a standard BCA method. SDS-PAGE was performed using standard techniques. Blots were blocked with 8% non-fat dry milk in TBST for 2 h at room temperature and subsequently incubated with primary antibodies in blocking solution overnight at 4 °C. The primary antibodies used were shown in Table S3. Primary antibodies were detected with either an anti-mouse or anti-rabbit HRP-conjugated secondary antibody (1:5000; Bio-Rad), and signals were developed using SuperSignal West Pico Chemiluminescent Substrate (Pierce). ImageJ was used to calculate the relative density of the protein. At least three independent western blots were conducted, and one representative blot is presented.

### Coimmunoprecipitation and quantification

Transfected 293T cells were harvested and lysed in lysis buffer (50 mM Tris-HCl, pH 7.5, 150 mM NaCl, 1% Nonidet P-40, 0.5% sodium deoxycholate and 1% protease inhibitor cocktails; Sigma, St. Louis, MO, USA) and incubated on ice for 5 min with periodic agitation. Cell lysates were centrifuged, and the supernatant was incubated with antibodies of interest and Protein G Plus/Protein A agarose beads (Sigma, St. Louis, MO, USA) or Flag M2-conjugated agarose beads (Sigma, St. Louis, MO, USA) at 4 °C overnight. The beads were washed six times with cell lysis buffer, and the precipitated proteins were further analyzed by western analysis.

To quantify the interaction between WT/mutant PITX2 and NRF2/ YAP1, ImageJ was used to calculate the intensity of the targeted protein. The intensity of IP bands/ input bands was calculated to normalize the total protein content. The relative binding capacity was quantified by the normalized IP-HA intensity/normalized IP-Flag intensity. At least three independent co-IP experiments were conducted, and one typical blot is presented.

### Measurement of superoxide production

Superoxide production was evaluated in retinal cryosections using dihydroethidium (DHE) as described previously [31]. Briefly, frozen sections were incubated with DHE (2 μM) for 30 min at 37 °C. DHE is oxidized upon reaction with superoxide to ethidium bromide, which binds to DNA in the nucleus and fluoresces red. Excessive reactive oxygen species (ROS) production is a hallmark of oxidative stress. Thus, retinal levels of superoxide, as determined by DHE staining with subsequent quantification of fluorescence intensity, was measured. Images were captured on a Zeiss LSM 800 confocal scanning microscope. The relative fluorescence intensity within the images obtained was determined via automated image analysis of ZEISS ZEN Intellesis or ImageJ software.

### Measurement of the MDA level and SOD, GSH-Px activity

Retinas were immediately extracted from enucleated eyes. The weighed retinal samples were prepared as a 10% homogenate in 0.9% saline. After homogenization on ice, the homogenate was sedimented at 2000 × *g* for 10 min, and the supernatant was collected and diluted. The GSH-Px, SOD activity, and MDA levels of the retinal lysates were determined using ELISA kits from the Nanjing Jiancheng Bio-company (Cat#A005, A001, A003, respectively, Nanjing, China). All procedures were carried out according to the manufacturer’s instructions.

### Statistical analysis

Statistical analysis was performed using GraphPad Prism 6 software. The data sets were tested for normal distribution using Shapiro–Wilk test. For normally distributed data, statistical significance was determined by Student’s *t*-test or ANOVA. If the data was not normally distributed, non-parametric statistic was used. *p*-values were calculated by Student’s *t*-test or ANOVA followed by a, Tukey, Dunnett or Sidak’s multiple comparisons test as appropriate. *P* < 0.05 was considered statistically significant.

## Results

### A new model of early-onset glaucoma identified in an ENU screening program

**Fig. 1:**
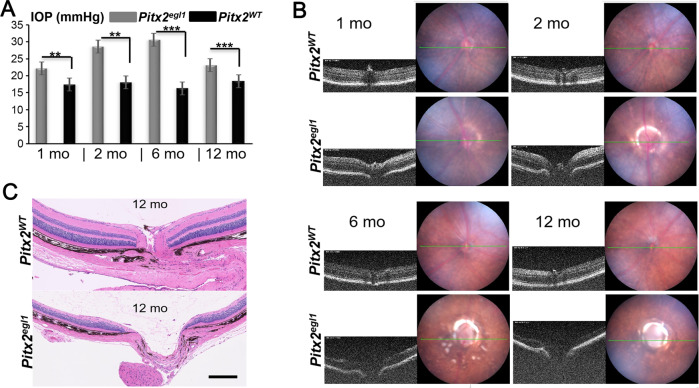
*Pitx2*^*egl1*^ mice exhibit an early-onset glaucoma phenotype. **A** Longitudinal intraocular pressure analysis of *Pitx2*^*WT*^ and *Pitx2*^*egl1*^ mice (*n* = 12 for each group). Intraocular pressure (IOP) measurements with a rebound tonometer (TonoLab) showed a progressive increase in pressure in *Pitx2*^*egl1*^ mutant mice. IOP (gray bar, *egl1* mice = 22.1 ± 3.4 and black bar, WT mice = 17.4 ± 2.2) at 1 month of age. IOP (gray bar, *egl1* mice = 28.6 ± 5.0 and black bar, WT mice = 18.0 ± 1.7) at 2 months of age. IOP (gray bar, *egl1* mice = 30.6 ± 6.5 and black bar, WT mice = 16.3 ± 1.3) at 6 months of age. IOP (gray bar, *egl1* mice = 23.1 ± 1.4 and black bar, WT mice = 18.4 ± 1.7) at 12 months of age. **B** Representative fundus photographs and optical coherence tomography images of *Pitx2*^*WT*^ and *Pitx2*^*egl1*^ mice at different ages. A partial ring around optic cup in the fundus and and optic nerve cupping observed by OCT in *Pitx2*^*egl1*^ mice (bottom row), but not in *Pitx2*^*WT*^ mice (upper rows) at 2, 6, and 12 months of age. The green lines indicate the plane in which the B-scan to the left of the fundus images were taken **C** Representative images of histologic sections taken through nerve heads from *Pitx2*^*WT*^ and *Pitx2*^*egl1*^ mice at 12 months of age. Optic nerve head cupping was observed in *Pitx2*^*egl1*^ mice, with thinning of inner retina layers. Scale bar: 200 μm. **P < 0.01; ***P < 0.001. Data are presented as the mean ± SEM.

**Fig. 2:**
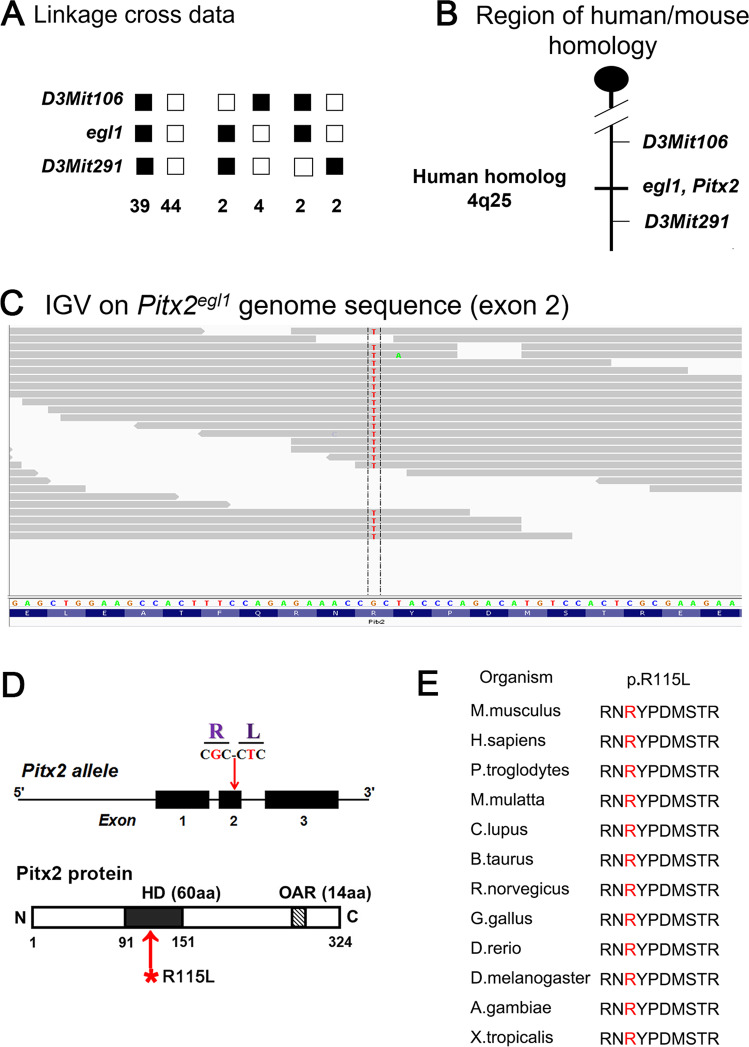
Identification of *Pitx2* mutation in *Pitx2*^*egl1*^ mice by whole-exome sequencing. **A** Genetic and molecular analysis of the *egl1* strain. A total of 93 progeny from a *egl1/egl1* X (*egl1*/DBA/2J) F1 backcross were genotyped in a genome-wide scan. Linkage to several markers on mouse Chr 3 was observed. The columns of squares represent haplotypes (filled boxes, *egl1/egl1* B6 alleles; open boxes, *egl1*B6/DBA/2J allele). The number of chromosomes associated with each haplotype is indicated below each column. **B** Genetic map of Chr 3 in the *egl1* region showing the closest markers and the region of human homology. **C** Integrative Genomics Viewer (IGV) shows the base substitution. **D** A homozygous missense mutation, c. G344T, p. R115L of the *Pitx2* gene was identified in *Pitx2*^*egl1*^ mice by whole-exome sequencing (WES). Upper panel: The schematic diagram shows the location of the missense mutation in the *Pitx2* gene. Bottom panel: Functional domains of PITX2 protein with the location of the mutations. HD, homeodomain; OAR, opt aristaless and rax. **E** Protein sequence alignment of amino sequences surrounding the *Pitx2* mutation with orthologous sequences from Mus musculus to Xenopus tropicalis. The mutant amino acid residue is in red. Note that the amino acid sequences surrounding the affected amino acid residues are also highly conserved.

The *egl1* mutant, with bulging eyes, was discovered while screening an ENU treated cohort for craniofacial phenotypes. Affected mice were distinguished from C57BL/6J controls by a high IOP phenotype (Fig. 1A). Optic neuropathy characterized by optic nerve cupping, and by severe retinal nerve fiber layer (NFL) loss in mutant retinas (Fig. 1B) was detected with optical coherence tomography. Histological study of retinas from 12-month-old mice confirmed the clinical assessment, and revealed enlarged optic disc cup with thinning of the inner retinal layer in mutant mice (Fig. 1C). Whole-exome sequences (WES) revealed a G to T base-pair transversion in *Pitx2* (Fig. 2A–D). Allele-specific PCR (Fig. S1) and Sanger sequencing (Fig. S2) further confirmed the presence of *Pitx2*^*egl1*^ mutation in a segregating cross. Thus, a novel homozygous missense mutation, c. G344T, p. R115L (NM_001042502.2), in the *Pitx2* gene was identified and deemed the most likely candidate for the glaucoma-like symptom in *egl1/egl1* mice. Notably, the affected amino acid residue is located in the HD region of PITX2 protein (Fig. 2D, lower panel) and is highly conserved across species (Fig. 2E).

### Generation of the *Pitx2*^*Mut*^ mouse model

In order to confirm the causative nature of the *egl1* mutation and explore the *Pitx2* mutation further, a *Pitx2*^*R115L*^ knock-in mouse model (named KI) was generated using CRISPR/Cas9 technology (Fig. S3). Homozygous *Pitx2*^*KI*^ mice were born at the expected Mendelian ratio (47 out of 220 in *Pitx2*^*KI/+*^ to *Pitx2*^*KI/+*^ crosses) and was not significantly different from the expected, 55 out of 220 mice. No significant changes in the protein content and localization pattern of PITX2 were observed in mutant retina compared to that of control (Fig. S3C, D). Immunofluorescent staining of sections from WT mice indicated that PITX2 was strongly expressed in the mouse GCL, where it colocalized with the RGC marker, Brn3a (Fig. S3D, upper panel), suggesting that PITX2 may play a role in RGC function.

### *Pitx2*^*KI*^ mice develop elevated IOP subsequent to anterior segment dysgenesis

**Fig. 3:**
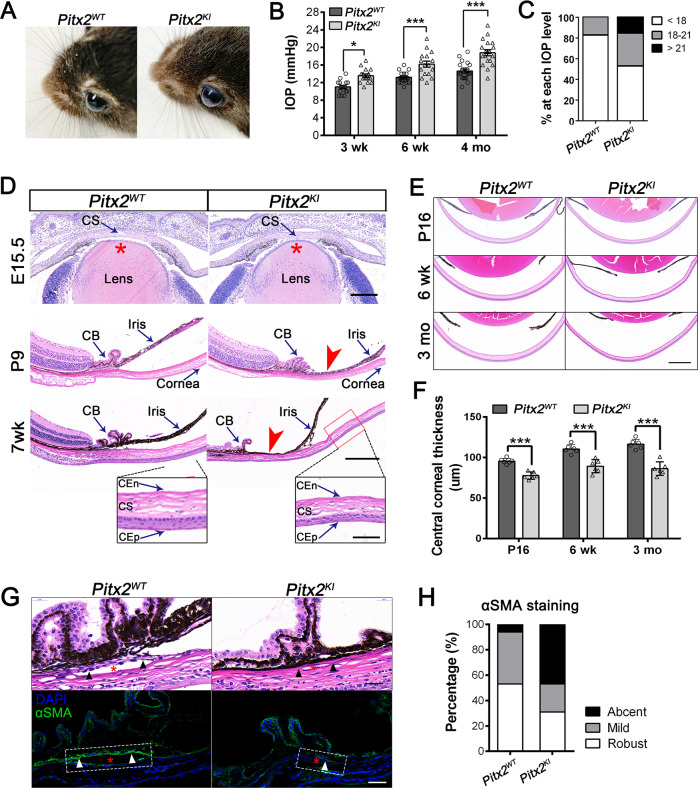
Elevated intraocular pressure and hypoplasia of ocular anterior segment in *Pitx2*^*KI*^ mice. **A** Image of *Pitx2*^*WT*^ and *Pitx2*^*KI*^ adult mice at 4 months of age. **B** Intraocular pressure analysis in the *Pitx2*^*WT*^ and *Pitx2*^*KI*^ mice at 3 weeks (*n* = 16), 6 weeks (*n* = 16) and 4 months (*n* = 18) of age. Individual IOP values were shown as dots in the histogram. **C** Histogram showing the proportion of eyes in each IOP range at 4 months. **D** Hematoxylin and eosin (H&E) staining of eyes of *Pitx2*^*WT*^ and *Pitx2*^*KI*^ mice at E15.5, P9, and 7 weeks. Compared with *Pitx2*^*WT*^ mice, anterior-segment development in E15.5 *Pitx2*^*KI*^ mice was slightly impaired. Scale bars: 100 μm. Iris-cornea adhesion and closed iridocorneal angle (red arrows) were detected in mutant mice at P9 and 7 weeks of age. Scale bars: 200 μm. The bottom panels show high-magnification images of the boxed areas, which denotes a thinner corneal epithelium in *Pitx*^*KI*^ mice at 7 weeks. Scale bars: 50 μm. CB, ciliary body; CEn, corneal endothelium; CEp, corneal epithelium; CS, corneal stroma. **E** H&E-stained sagittal sections of E15.5, P9, and 7 weeks old WT littermates and mutant samples. Scale bars: 200 μm. **F** A comparison of central corneal thickness between mutants and their WT littermates (*n* = 6 in each group). Mutant mice present a thinner central cornea relative to their WT littermates. Individual thickness values are shown by dots in the histograms. *P < 0.05; ***P < 0.001. Data are presented as mean ± SEM. **G** Position-matched images of the H&E staining (upper panel) and the immunofluorescent staining (lower panel) of optic sagittal sections are shown. Trabecular meshwork (TM) is labeled with alpha-smooth muscle actin (α-SMA), and nuclei counter-stained with DAPI. Conventional outflow tissues are outlined by white boxes. Asterisks indicate Schlemm’s canal (SC) lumen, arrowheads show trabecular meshwork. Scale bars: 25 μm. **H** Data show the percentage of α-SMA staining measurements in the boxed TM region. Staining was assessed by ratings of “robust”, “mild”, or “absent”. (*n* = 6 in each group). *P < 0.05; ***P < 0.001. Data are presented as mean ± SEM.

Similar to the *Pitx2*^*egl1*^ mutant mice, we observed that approximately 59% of mutants (20 out of 34) exhibited a bulging and distended appearance of the eye with a mild corneal opacity (Fig. 3A), as early as 2 months of age. Moreover that roughly 80% of these cases were bilaterally affected, as reported for ARS patients [19]. Rebound tonometry confirmed elevated IOPs in mutants. As expected, *Pitx2*^*KI*^ mice exhibited a progressive elevation of IOP measurements, 13.87 ± 0.89 mmHg (*n* = 16), 16.38 ± 1.13 mmHg (*n* = 16), and 18.78 ± 1.54 mmHg (*n* = 18) at 3, 6 weeks, and 4 months of age, respectively. In contrast, the IOP measuring of their control littermates at corresponding ages were 11.16 ± 1.58 mmHg (*n* = 16), 13.17 ± 1.88 mmHg (*n* = 16), and 14.62 ± 2.34 mmHg (*n* = 18) (Fig. 3B), which is consistent with previous measurements for C57BL/6Jstrains [32]. By 4-months of age, 47% of mutants presented with high IOP levels of >18 mmHg, and ~14% mutants with IOP > 21 mmHg, a level commonly associated with glaucoma (Fig. 3C). Interestingly, nearly 53% of mutants did not develop ocular hypertension at this stage indicating incomplete penetrance of the disease. Similarly, only 50% of ARS patients develop elevated IOP and glaucomatous pathologies [33–35].

Previous studies have demonstrated that PITX2 is required for normal ocular development [21, 36]. We, therefore, sought to determine whether the elevated IOP in *Pixt2*^*KI*^ mice was associated with aberrant ocular development. Mice were examined at E15.5, P9 and 6 weeks for morphological alterations by histology. The anterior chamber of mutant mice at E15.5 appeared slightly collapsed and showed delayed separation of the cornea from the lens surface compared to *Pitx2*^*WT*^ mice, but otherwise no other suggestion of abnormal development was apparent (Fig. 3D, upper panel). By contrast at P9, multiple malformations of the anterior chamber were observed in mutant eyes. A distinct disruption of the iridocorneal angle with adhesion of the iris to cornea, creating a fully angle closure was observed in mutant eyes (Fig. 3D, middle panel). The iris-cornea adhesion was more pronounced in adults, at 7 weeks of age, and was accompanied by iris hypoplasia and ciliary body atrophy in all mutant eyes (Fig. 3D, lower panel).

Moreover, histological analysis in 7-week-old *Pitx2* mutant mice revealed a thinner cornea compared to that of control, with a reduced thickness of both the epithelial and stromal layers (Fig. 3D). Detailed longitudinal analysis of the mutant and WT mice from P16, 6-week- to 3-month-of age confirmed the reduced central corneal thickness (Fig. 3E, F), suggesting an abnormal corneal development. In addition, the TM and Schlemm’s canal (SC) in 1-month-old mutants were occluded and not readily identifiable, and the trabecular endothelial cells were tapered and attenuated without regular cellular components (Fig. 3G, upper panel). To further examine the TM malformation, ocular frozen sections were stained for α-SMA, a marker for TM cells. This analysis revealed that the majority of mutants exhibited an absence or smaller area of α-SMA staining compare with that of control, corroborating the TM hypoplasia in *Pitx2*^*KI*^ mice (Fig. 3G, H). It should be noted that all heterozygotes (*Pitx2*^*KI/+*^) tested at 3 months of age were not significantly different in ocular morphology or in IOP levels compared to their WT littermates (Fig. S4A, B), and all retinal structures, including the optic nerve head and anterior segment appeared normal (Fig. S4B, C).

### Retinal ganglion cell loss and optic nerve degeneration in *Pitx2*^*KI*^ mice

To determine whether high IOP leads to optic neuropathy in *Pitx2*^*KI*^ eyes, we assessed retinal and optic nerve morphology in histological sections ON. Eyes of P16 mutant mice, an age prior to IOP elevation, exhibited a normal retinal layer morphology (Fig. S5A). At 6 weeks, a mild loss of cells in the ganglion cell layer (GCL) was observed in mutant mice (Fig. S5B), and this trend generally progressed with age.

**Fig. 4:**
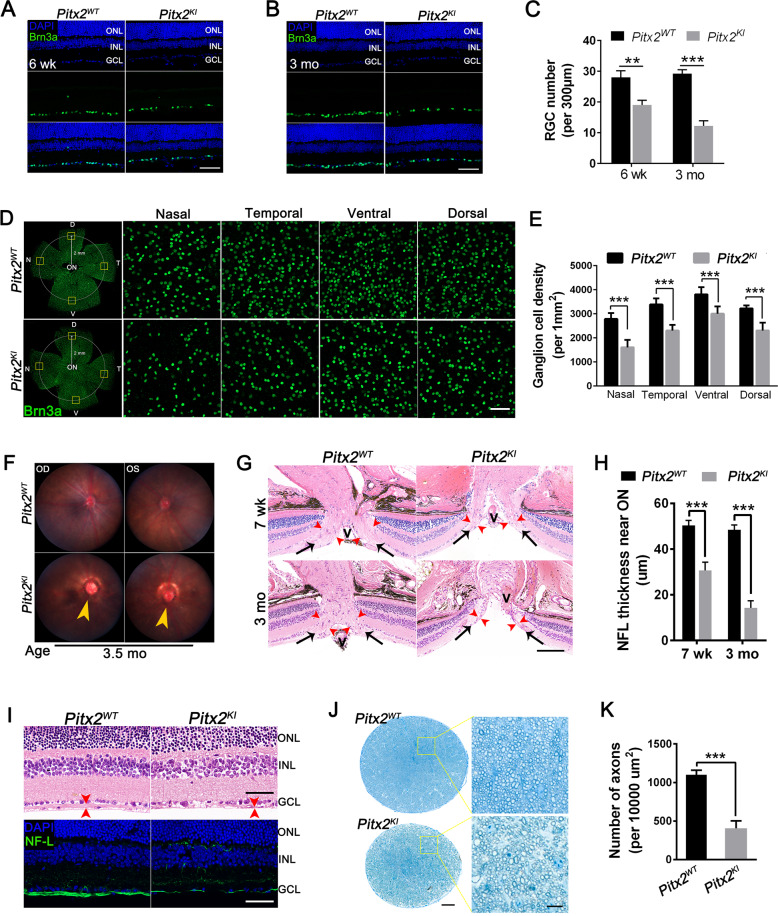
Ganglion cell loss and optic nerve atrophy in *Pitx2*^*KI*^ mutant mice. **A**, **B** Retinal RGCs from 6-week-old (**A**) and 3-month-old (**B**) mice were labeled with anti-Brn3a, and the nuclei were counter-stained with DAPI. Scale bars: 25 μm. **C** Quantification of the Brn3a^+^ RGC number in GCL. *Pitx2*^*KI*^ mutant mice at 3 months of age exhibit slightly thinner inner and outer nuclear layers compared with WT mice (*n* = 6 per cohort). **D** Representative immunofluorescent enface images of enface view of a retinal flatmount from WT and mutants at 3 months of age labeled with anti-Brn3a for detecting RGCs. Scale bars: 50 μm. **E** *Pitx2*^*KI*^ mutants have significantly decreased numbers of RGCs per mm^2^ (*n* = 8) relative to their control littermates (*n* = 10). **F** Fundus photograph at 5 months shows abnormal morphology of optic nerve head (arrowhead) in the eyes of *Pitx2*^*KI*^ mutant mice. **G** Representative images of histologic sections taken through nerve heads from *Pitx2*^*WT*^ and *Pitx2*^*KI*^ mice at 7 weeks and 3 months of age. The optic nerve head appeared abnormal in *Pitx2*^*KI*^ mice at both ages, with thinning of the nerve fiber layer (NFL) (red arrowheads) and optic nerve excavation is noted (asterisk). Black arrows indicate NFL entering the optic nerve (ON); V: Central blood vessel. Scale bars: 200 μm. **H** Quantitation and comparison of the NFL thickness near the optic nerve in 7-week and 3-month-old mice (*n* = 6 per cohort). **I** Position-matched images of the H&E staining (upper panel) and the immunofluorescent staining (lower panel) of retinal cross sections are shown. Retinal nerve fibers were labeled with neurofilament-L (NF-L), and the nuclei were counter-stained with DAPI. Scale bars: 25 μm. **J** Optic nerve atrophy was assessed in cross semithin sections of resin-embedded optic nerves from 5-month-old WT and mutant mice stained with toluidine blue staining. Scale bars: 100 μm. The panels on the right show high-magnification images of the boxed areas shown in the panels on the left. Scale bars: 10 μm. **K** Quantification of axon number in the optic nerve of 5-month-old mice (*n* = 10 per cohort). The *Pitx2*^*KI*^ mutants have significantly less axonal projections compared to *Pitx2*^*WT*^ mice. ONL, outer nuclear layer; INL, inner nuclear layer; GCL, ganglion cell layer. The statistics were calculated based on data from all tested mutants. The representative images of *Pitx2*^*KI*^ were from mutants with elevated IOPs. **P < 0.01; ***P < 0.001. #, no significant difference. The data are presented as mean ± SEM.

At 3 months, mutant mice showed a mild thinning of outer and inner nuclear layers (Fig. S6A, B), accompanied by a 61.2% decrease in GCL cell number compared to that of controls (Fig. S6A, C), indicating significant RGC loss. This result was precisely confirmed with anti-Brn3a, an RGC-specific marker, stained retinal section from 6-week-old (Fig. 4A) and 3-month-old (Fig. 4B) mice, which demonstrated a progressive RGC death in mutants (Fig. 4C). RGC density was further examined with anti-Brn3a stained retinal flatmounts from 3-month-old animals. Each anti-Brn3a stained retinal flatmount was segmented into nasal, temporal, ventral, and dorsal quadrants, and RGC density quantified respectively. In mutant retinas, a significant decrease in Brn3a-positive cells in all four quadrants was observed (Fig. 4D). Quantitative analysis revealed a 43.4 %; 32.8%; 24.3%, and 27.5% reduction of Brn3a-positive cells compare to that of WT in nasal, temporal, ventral, and dorsal area, respectively (Fig. 4E).

We next evaluated optic disc cupping, a characteristic pathology of glaucoma. Clinical fundus examination revealed a glaucomatous fundus appearance with an asymmetric and severely excavated ON head and peripapillary chorioretinal atrophy in mutant eyes (Fig. 4F). Consistent with clinical fundus examinations, histological analysis revealed optic disc cupping and thinning of the NFL near the ON in mutant mice at 7 weeks of age, and these phenotypes became more severe at 3 months (Fig. 4G, H). Moreover, severe axon loss was also visible in the central retina of mutant mice, as evidenced by an extremely atrophied NFL, which was confirmed by Neurofilament-L staining (Fig. 4I). To determine details of axonopathy, toluidine blue-stained semithin sections of the ON from 3-month-old animals were prepared and the number of axons were quantified. Compared with WT mice, the axons density was clearly lower in mutant mice and was accompanied by axonal swelling and extensive myelin debris (Fig. 4J, K), indicating severe axonal damage. Additionally, to assess if pathological changes in mutants with normal IOPs (≤18) occurred, we also examined these mutants. Interestingly, pathologic features of glaucomatous injuries, such as RGC loss (Fig. S7A, B) and ON damage (Fig. S7C–E). were observed but the phenotypes appeared milder compared to mutants with elevated IOPs.

Vision loss after ON degeneration is also a hallmark of glaucoma. We thus tested vision function of 3-month-old mice using ERG recordings. The scotopic b-wave amplitude was reduced at 0.3, 3, and 20 cd sec/m^2^ flash intensities (Fig. S8A–C, G), and the photopic b-wave (Fig. S8D, E, H) and flicker amplitude (Fig. S8F, I) was also reduced in mutant mice, while no difference was observed for the scotopic a-wave amplitude (Fig. S8A–C, G), suggesting impaired visual function in the inner retina. Additionally, the amplitudes of the oscillatory potentials (OPs), which originate from the functional inner retina in mice [37], were also significantly reduced in mutant mice (Fig. S9), reflecting inner retinal dysfunction.

### Biochemical analysis of mutant PITX2 proteins

**Fig. 5:**
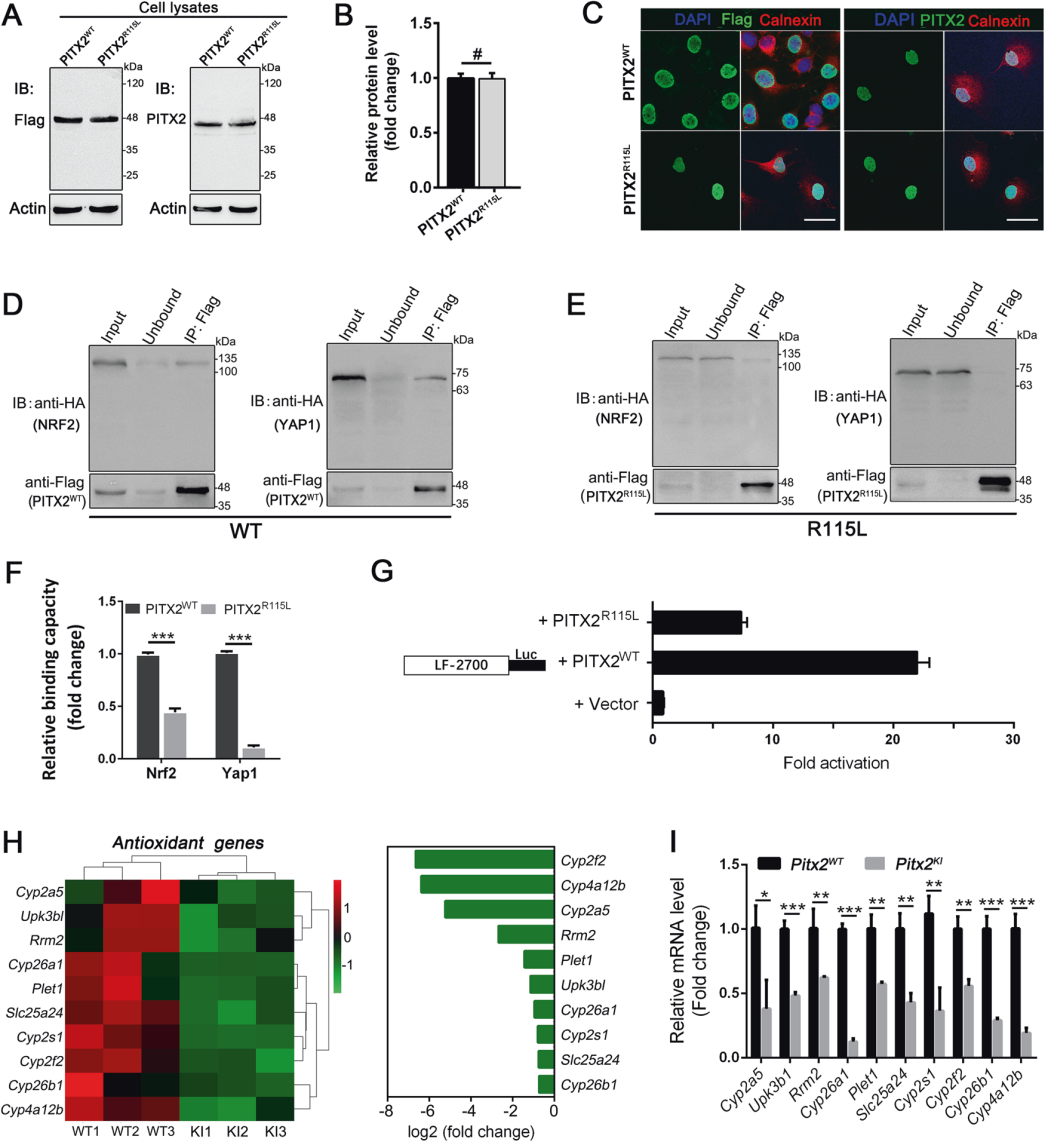
Analysis of the biochemical and molecular changes observed in *Pitx2*^*KI*^ mutant mice. **A** Expression of WT and mutant PITX2 proteins in 293T cells. The *pCMV-Pitx2*^*WT*^ and *pCMV-Pitx2*^*R115L*^ expression plasmids were transfected into 293T cells. Western blot analysis showed mutant PITX2 protein had the same abundance as WT. Antibody of Flag-tag was used to verify the specificity of PITX2 antibody. β-actin was used as the loading control. **B** Quantitative comparison of the WT and mutant PITX2 protein levels in transfected cells (*n* = 6 per cohort). **C** Subcellular localization of WT and mutant PITX2 proteins in COS7 cells. Immunocytochemistry study using anti-PITX2 antibody indicated that both WT and mutant PITX2 proteins were localized in the nucleus. **D** 293T cells were co-transfected with the indicated constructs. Flag immunoprecipitation, and blotting with anti-HA confirmed physical interaction. PITX^WT^-Flag was immunoprecipitated and the amount of co-precipitated NRF2-HA (left panels) or YAP1-HA (right panels) was determined by immunoblotting, shown are representative immunoblots. **E** PITX2^R115L^-Flag was immunoprecipitated and the amount of co-precipitated NRF2-HA (left panels) or YAP1-HA (right panels) was determined by immunoblotting, shown are representative immunoblots. **F** Quantification of immunoblots reveals the PITX2 p. R115L mutation decreases its interaction with NRF2 and YAP1 compared to WT (*n* = 4 in each group). **G** The transcriptional activity of WT and mutant PITX2 was detected with a luciferase reporter in transient transfections of 293T cells. All luciferase activities are normalized to β-galactosidase activity and shown as mean-fold activation compared with the vector. Five independent replicas were performed. The mean LEF-1 promoter luciferase activity with PITX2 expression was about 200,000 light units per 15 μg protein, and the β-galactosidase activity was about 65,000 light units per 15 μg protein. **H** RNA-seq results showing that several antioxidant-related genes are downregulated in mutant retinas. **I** RT-qPCR verified the RNA-seq results for the indicated genes (*n* = 12). *P < 0.05; **P < 0.01; ***P < 0.001. #, no significant difference. The data are represented as mean ± SEM.

RT-PCR was performed on mRNA extracted from retina of adult WT mice. Among the three alternatively-spliced murine isoforms (*Pitx2a*, *Pitx2b* and *Pitx2c*) (Fig. S10A), RT-PCR analysis revealed that only *Pitx2c* isoform was expressed in the mouse retina (Fig. S10B). c. G344T variant was introduced into *Pitx2c* coding sequence by site-directed mutagenesis to examine the impact of this variant. Western analysis showed unique immune positive bands when probed with anti-PITX2 antibody or Flag-tag antibody (Fig. 5A), suggesting unchanged expression level (Fig. 5B). Immunofluorescence staining revealed that both WT and mutant proteins mainly localized in the nucleus (Fig. 5C). These data demonstrated that this variant is unlikely to impact the expression and cellular localization of the PITX2 protein.

### The missense mutation disrupts the interaction of PITX2 with NRF2/YAP1 and impairs the transcriptional activity of PITX2 for antioxidant genes

**Fig. 6:**
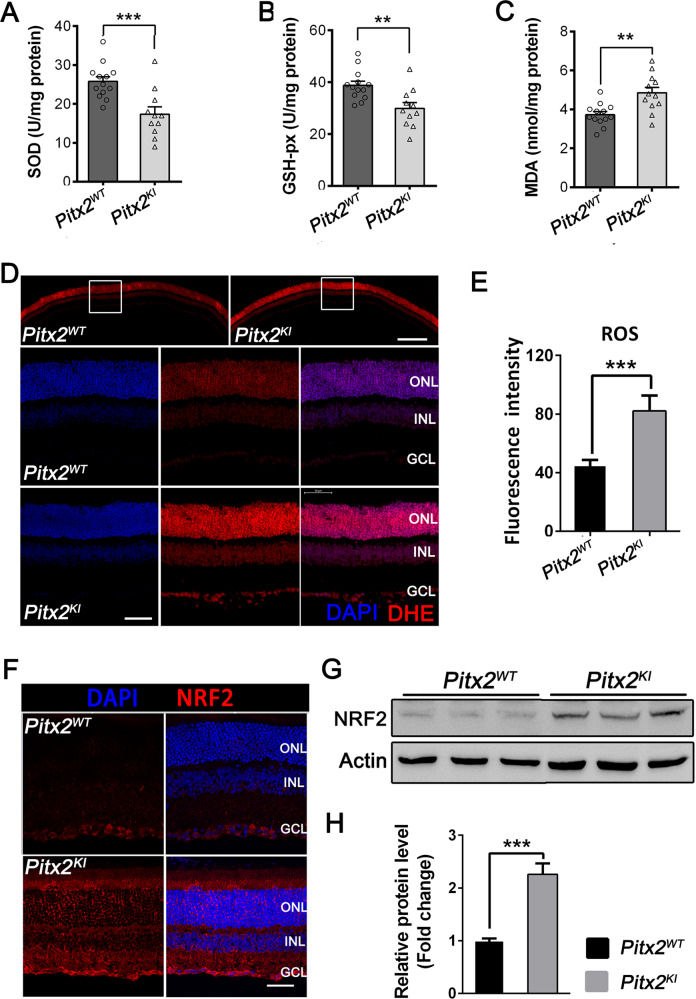
Increased oxidative stress in the retinas of *Pitx2*^*KI*^ mice. **A**–**C** Mean levels of SOD (**A**) and GSH-Px (**B**) activities, MDA (**C**) content in the *Pitx2*^*WT*^ (*n* = 13) and *Pitx2*^*KI*^ (*n* = 11) retinas. Individual values are shown by dots in the histogram. **D** ROS activity measured by DHE staining; the red staining is positive for ROS content. Nuclei were counter-stained with DAPI (blue). Scale bars: 200 μm. High-magnification images of boxed areas are shown in the lower panel. Scale bars: 25 μm. **E** Quantification of the fluorescence intensity of DHE staining (*n* = 9). **F** Representative confocal microscopy images of immunofluorescent staining of NRF2 (oxidative stress regulator, red) and DAPI (blue) in retina sections from 5-week-old mice. Scale bar: 25 μm. **G** Representative immunoblots of 5-week-old retinal lysates from *Pitx2*^*WT*^ and *Pitx2*^*KI*^ mice stained for NRF2. β-actin was used as the loading control. Uncropped immunoblotting images are shown in Fig. S12. **H** Quantification of immunoblots of retina lysates from *Pitx2*^*WT*^ and *Pitx2*^*KI*^ mutant mice. NRF2 level (normalized to control) in retinas from mutant mice was elevated compared with levels in control mice (*n* = 9). ONL, outer nuclear layer; INL, inner nuclear layer; GCL, ganglion cell layer. *P < 0.05; **P < 0.01; ***P < 0.001. The data are presented as mean ± SEM.

Previous studies suggest PITX2 expression and activity depends on NRF2-activated transcription and nuclear shuttling, where PITX2 binds to YAP1 and cooperatively activates transcription of antioxidant genes in mouse myocardium after injury [38]. We reasoned that the interactions of PITX2 with NRF2/YAP1 could also play key roles in the transcription of antioxidant genes under injury condition in the retina. Thus, we analyzed the interactions between WT or mutant PITX2 and NRF2/YAP1 by coimmunoprecipitation, in transiently transfected 293T cells. Upon co-expression of Flag-tagged WT PITX2 and HA-tagged NRF2, a distinct anti-HA signal (NRF2) was detected in cell lysates as well as Flag precipitates (Fig. 5D, left panel), confirming that PITX2 interacts with NRF2. We similarly confirmed interactions of PITX2 with YAP1 (Fig. 5D, right panel). By contrast, in the presence of mutant PITX2, the anti-HA signal (NRF2) was significantly weakened in Flag precipitates using the same procedure (Fig. 5E, left panel), and that of YAP1 was almost undetectable (Fig. 5E, right panel). Relative quantification (detail in methods) indicated that the binding capacity of mutant PITX2 to NRF2 and YAP1 was reduced 53.78% and 89.12% of WT PITX2, respectively (Fig. 6C), indicating that the missense mutation disrupts the interaction of PITX2 with NRF2 and YAP1, which might in turn affect its transcriptional activity. We then evaluated the transcriptional activity of the mutant PITX2 by assessing luciferase activity in transfected 293T cells using the LF-2700 promoter constructs [29]. Compared with the empty vector, WT PITX2 activated transcription from the LF-2700 promoter construct by approximately 12-fold (Fig. 5G). By contrast, the mutant PITX2 lost ~69.7% of its transcriptional activity compared to WT version (Fig. 5G). Thus, the missense mutation impairs the ability of PITX2 protein to recognize and bind to its target sequences and activate transcription of downstream genes. Additionally, to further determine whether the NRF2-YAP1 axis participates in anterior chamber failure in *Pitx2*^*KI*^ animals, ocular cryosections were immunofluorescently labeled using antibodies against NRF2 and YAP1. No significant difference in staining was observed between WT and mutant mice (Fig. S11), implying that other mechanisms may underly the anterior chamber malformations.

We next performed transcriptomic analysis using of retinas from 2-month-old *Pitx2* mutant mice and WT littermate controls to obtain insights into the molecular mechanisms. Analyses of the RNA-seq data revealed that expression of the antioxidative response-related genes (*Cyp2a5*, *Upk3b1*, *Rrm2*, *Cyp26a1*, *Plet1*, *Slc25a24*, *Cyp2s1*, *Cyp2f2*, *Cyp26b1*, and *Cyp4a12b*) were significantly downregulated in *Pitx2* mutant retinas (Fig. 5H). These results were further confirmed by real-time quantitative PCR (qPCR) (Fig. 5I). Taken together, based on the transcriptome results, we propose that the rapid RGC loss and ON dystrophy in mutant retina results not only from the elevated IOP caused by anterior segment dysgenesis, but also to the compromised antioxidant capacity caused by impaired PITX2 transcriptional activity of antioxidant genes.

### *Pitx2*^*KI*^ mutant retinas exhibit increased oxidative stress

Given the impaired PITX2-mediated transcription of antioxidant genes in mutant mice, we next examined the oxidative status of *Pitx2*^*KI*^ retinas of 6-week-old mice. Several indicators of oxidative stress were evaluated by ELISA using isolated retinal lysates. Compared to controls, decreased activities of the antioxidant enzymes superoxide dismutase (SOD) and glutathione peroxidase (GSH-Px) and increased malondialdehyde (MDA) content were observed in mutant retinas (Fig. 6A–C), suggesting compromised antioxidant capacity and peroxidation in mutant retinas.

Retinal cryosections stained with dihydroethidium (DHE) (Fig. 6D) showed increased DHE reaction in n mutant retinas. Quantification of fluorescence intensity revealed a significant increase in superoxide production in mutant mice, compared to that of controls (Fig. 6E). In addition, both immunofluorescence and western blot demonstrated a significant increase in the NRF2 level in mutant retinas compared to controls (Fig. 6F–H). NRF2 regulates the expression of antioxidant proteins that attenuate cellular oxidative stress, thus its increased expression signifies activation of oxidative stress.

### Glial activation and RGC apoptosis in *Pitx2*^*KI*^ retinas

We assessed the status of glial activation in mutant retinas from 2-month-old mice. In control retinas, CD68 immunoreactivity was weak and punctate, while in mutant retinas staining was intense and agminated in the inner retina (Fig. S12A), suggestive of an activated state. Moreover, the expression level of another microglia marker Iba1 was found to be increased in mutant retinas by western blot analysis (Fig. S12B, C). Additionally, activated Müller glia were also distinctly recognizable in mutant retinas by immunostaining with an antibody against glial fibrillary acidic protein (GFAP) (Fig. S12A). These data confirmed astrogliosis in mutant retinas characterized by upregulation of GFAP level relative to controls (Fig. S12B, C). TUNEL assay revealed increased cell apoptosis in the GCL of mutant retinas, likely due to prolonged oxidative stress and excessive inflammatory reaction (Fig. S12D, E).

### Treatment with N-Acetyl-L-cysteine mitigates high intraocular pressure-induced retinal injury by inhibiting oxidative stress in *Pitx2*^*KI*^ retinas

**Fig. 7:**
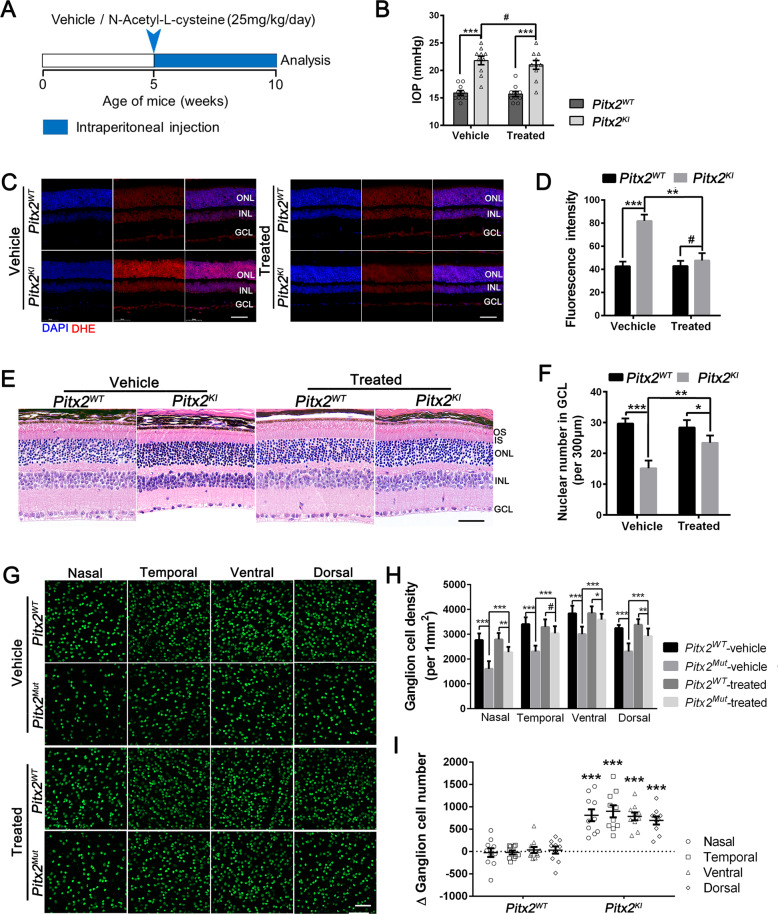
Treatment with N-Acetyl-L-cysteine prevents elevated intraocular pressure-induced retinal injury by inhibiting oxidative stress in *Pitx2*^*KI*^ mutant retinas. **A** N-Acetyl-l-cysteine in 40% PBS was injected intraperitoneally (25 mg/kg/day) into *Pitx2*^*WT*^ and *Pitx2*^*KI*^ mutant mice for 5 weeks (from 5-week to 10-weeks of age) as the treated groups. Equivalent volumes of 40% PBS were delivered intraperitoneally to the *Pitx2*^*WT*^ and *Pitx2*^*KI*^ mice as the vehicle-treated control groups. All animals were sacrificed at 10 weeks of age to conduct analyses. **B** IOP analysis in *Pitx2*^*WT*^ and *Pitx2*^*KI*^ mice subjected to vehicle and N-Acetyl-l-cysteine treatment. Individual IOP values are shown by dots in the histograms (*n* = 10 in each group). **C** ROS activity was measured by DHE staining of retinal sections from *Pitx2*^*WT*^ and *Pitx2*^*KI*^ mutant mice subjected to vehicle or N-acetyl-l-cysteine treatment. The red staining is positive for ROS content. Nuclei were counter-stained with DAPI (blue). Scale bars: 50 μm. **D** Quantification of the fluorescence intensity of DHE staining showed an obvious decrease in ROS formation in retinas from treated-*Pitx2*^*KI*^ mutant mice (*n* = 10 in each group). **E** Representative images of H&E staining of retinal section from *Pitx2*^*WT*^ and *Pitx2*^*KI*^ mice subjected to vehicle or N-Acetyl-l-cysteine treatment. Scale bars: 25 μm. OS, outer segment; IS, inner segment; ONL, outer nuclear layer; INL, inner nuclear layer; GCL, ganglion cell layer. **F** Quantification of the nuclei number in GCL shows that the *Pitx2*^*KI*^ mutants have significantly decreased number of nuclei in the GCL (*n* = 6 in each group). **G** Immunofluorescent enface view of retinal flatmounts from vehicle- or N-Acetyl-l-cysteine-treated *Pitx2*^*WT*^ and *Pitx2*^*KI*^ mice following labeling with anti-Brn3a to detect RGC. Scale bars: 25 μm. **H** Quantification of the number of RGC showed overall decreased number of RGL Brn3a positve cells. However, comparison of *Pitx2*^*KI*^ vehicle group, *Pitx2*^*KI*^ treated group showed significantly more RGC remaining in N-Acetyl-l-cysteine-treated *Pitx2*^*KI*^ mice (*n* = 12 in each group). **I** Quantitation indicates that an obvious increase in ∆ RGC number in *Pitx2*^*KI*^ retinas (*n* = 10 in each group). ∆ RGC number = RGC number (Treated group)–RGC number (vehicle group). A positive number indicates an increase in RGC number after N-Acetyl-l-cysteine treatment. *P < 0.05; **P < 0.01; ***P < 0.001. #, not significant. The data are presented as mean ± SEM.

The apparent oxidative stress and subsequent ocular injury prompted us to investigate whether an oxidative reductant would have protective efficacy in *Pitx2* mutant mice. N-Acetyl-L-cysteine, an antioxidant, provides increased glutathione and exerts antioxidative protection. Thus, to rescue the RGC degeneration, N-Acetyl-L-cysteine (25 mg/kg/day) or PBS (serve as vehicle control), were intraperitoneally administered daily to *Pitx2* WT or mutant mice after 5-weeks of age (Fig. 7A). After 5 weeks of treatment, mice were sacrificed to analysis.

N-Acetyl-L-cysteine-treated mutant mice (hereafter treated) showed no significant differences in IOP from vehicle-treated mutant mice (hereafter vehicle) (Fig. 7B). However, ROS activity was significantly affected in retinas of treated mutant mice. Imaging of vehicle mutant retinas demonstrated an increased DHE reaction in the GCL, INL, and ONL (Fig. 7C), while the DHE-superoxide reaction was largely prevented in the treated mutant retinas, which showed comparable staining intensity to that in WT retina (Fig. 7D). N-Acetyl-L-cysteine administration likely improved the oxidative stress response induced by elevated IOP in mutant retinas.

**Fig. 8:**
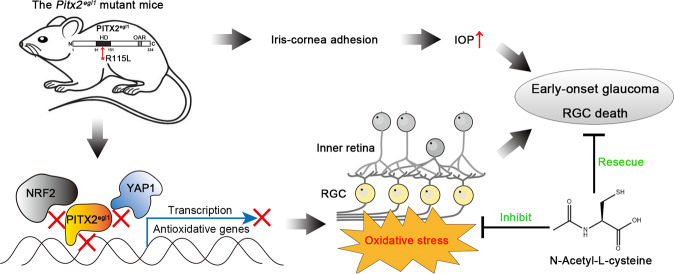
Schematic diagram of disease mechanism and therapy strategy for *Pitx2*^*egl*^ mutant mice. A novel missense mutation p.R115L in Pitx2 (named *Pitx2*^*egl1*^) led to iris-cornea adhesion and subsequent elevated intraocular pressure (IOP) accompanied with progressive death of retinal ganglion cells (RGC). These ocular phenotypes recapitulated hallmark features of the human glaucomatous pathologies. Mutant PITX2 protein loses its ability to bind NRF2 (regulated PITX2 expression and nuclei localization) and YAP1 (co-initiate transcription of their downstream targets) and consequently, PITX2-mediated transcription of several antioxidant genes is impaired, hence increasing oxidative stress in the inner retina. Treatment with N-Acetyl-l-cysteine exerted a profound neuroprotective effect on glaucomatous neuropathies associated with the model through inhibition of oxidative stress.

The RGC loss and ON damage was also evaluated by histological analysis. In vehicle mutant mice, the number of cells in GCL was drastically decreased compared to vehicle-treated WT controls. By contrast, the number of cells retained in the GCL were significantly greater in mutant mice administered N-Acetyl-L-cysteine, compared with corresponding vehicle-treated mice (Fig. 7E, F). The RGC density was further evaluated by immunofluorescent staining of Brn3a of retinal flatmounts. Consistent with H&E staining, N-Acetyl-L-cysteine treatment appeared to increas the number of Brn3a-positive cells in mutant retina compared to that of vehicle mutant mice in all four retinal quadrants (Fig. 7G), but still did not reach WT levels (Fig. 7H). To directly monitor the therapeutic effect on RGC death, the RGC number between vehicle- and treated-group of each genotype (indicated as ∆ RGC number) were quantified. An increased number of Brn3a-positive cells after N-Acetyl-L-cysteine treatment was observed (Fig. 7I). Together, these data suggested that N-Acetyl-L-cysteine displays significant therapeutic effect on glaucomatous neuropathies through mitigation of oxidative stress and subsequent partial preservation of RGC in *Pitx2*^*KI*^ retinas (Fig. 8).

## Discussion

The anatomical and physiological similarity between human and mouse eyes and the ability to genetically manipulate mice make them an excellent model to investigate disease mechanisms and potential intervention of diseases, such as glaucoma. The heritable DBA/2J glaucoma model has provided invaluable information on disease cell biology and intervention [39–41]. However, elevated IOP and glaucoma phenotypes do not manifest until 9–12 months, making mechanistic studies and testing of therapeutic strategies expensive and time-consuming. As a novel ENU induced *Pitx2*^*egl1*^ and the CRISPR/Cas9 mediated *Pitx2*^*KI*^ glaucoma models exhibit early-onset glaucoma, including elevated IOP and RGC degenerative phenotypes., they provide valuable insight into degeneration of RGC and its related molecular pathways. At the molecular level, the PITX2 p.R115L missense mutation disrupts binding of PITX2 to YAP1 and NRF2 and impairs its transcriptional activation of downstream genes involved in regulating oxidative stress (Figs. 5 and 6). Reduced activities of the antioxidant enzymes SOD and GSH-Px were observed in *Pitx2* mutant retinas, indicating compromised antioxidant capacity and peroxidation (Fig. 5). In support of this, treatment with N-Acetyl-l-cysteine mitigates the RGC injury observed, presumably by inhibiting oxidative stress in mutant retinas (Fig. 7).

Alterations in the level of functional PITX2 protein (either increased or decreased) may contribute to the pathologies observed [42]. Previous studies reported that heterozygote *Pitx2* null mice exhibited thinning of the ventral body, small body size, and ocular and tooth defects [21], whereas in other studies heterozygote alleles do not show obvious haploinsufficient phenotypes [43]. In the present study, heterozygous *Pitx2*^*KI*^ mutants do not show any obvious glaucomatous phenotype (Fig. S3). This is probably due to the fact that the missense mutation studied here is likely to be a hypomorphic variant (Fig. 5). Additionally, incomplete penetrance was evident in our mouse models, with only 47% of mutants presenting with elevated IOP at 4 months of age (Fig. 3C), which is consistent with the observation that only 50% of patients with ARS develop elevated IOP and glaucoma. Moreover, while teeth hypodontia or microdontia is reported in some human ARS patients and not others, no apparent non-ocular abnormalities were observed in adult *Pitx2*^*KI*^ mice. Thus, our mutants model recapitulates ocular phenotypes of ARS patients with *PITX2* mutations [16, 44, 45].

We demonstrate two possible pathological mechanisms that underlie the glaucoma phenotypes induced by the missense mutation in *Pitx2* (Fig. 8). The observed anterior segment dysplasia in *Pitx2*^*KI*^ mice likely contributes to the dysfunction of the drainage system leading to an elevation in IOP, which serves as an initiator of the disease process. The mutation also exacerbates the high IOP-induced oxidative stress in the retina by disrupting the interaction of PITX2 with NRF2/YAP1, which consequently impairs the transcription of several antioxidant genes. The reduction of antioxidant capacity may accelerate RGC loss and optic neuropathy. The anterior segment dysplasia caused by *Pitx2* variation has been well documented, but the precise mechanism through which subsequent pathogenesis occurs remains unclear. The present study mainly focuses on the latter, revealing a novel function of PITX2 in RGCs and in glaucomatous pathogenesis. We demonstrated that PITX2 moderates oxidative stress response through the NRF2-YAP1 axis in RGCs, whereas according to immunostaining data, this mechanism does not appear to be involved in the early post-natal phenotype (Fig. S11). The potential mechanisms triggering the anterior segment dysgenesis warrant further investigation.

Owing to the high demand of oxygen and energy necessary to generate action potentials, RGCs contain large number of mitochondria throughout the cell soma, axon and dendrites [46, 47]. Moreover, mitochondrial dysfunction has been recognized to be relevant to the development of glaucoma [48–52]. Transcriptional analysis of DBA/2J glaucoma model revealed mitochondrial dysfunction and metabolite depletion as a primary cause of neuronal damage in glaucoma [53]. These studies suggested that mitochondria dysfunction and oxidative stress play important roles in glaucoma. In our *Pitx2*^*KI*^ early-onset glaucoma model, elevated expression levels of NRF2, a regulator of cellular resistance to oxidative stress, was observed (Fig. 6F–H). N-acetyl-L-cysteine treatment mitigated RGC loss by inhibiting oxidative stress in *Pitx2* mutant retinas (Figs. 7 and 8). Cumulatively, our data identifies a potential target for development of therapeutic strategies for ARS induced glaucoma and also, a potential strategy for glaucoma therapy development.

## Supplementary information

Supplemental data

Supplemental Table 1

Supplemental table 2

Uncropped gel images

## Data Availability

All data generated or analyzed during this study are included in this published article and its supplementary information files. The data sets analyzed during the current study are available in Repository with Accession ID: CRA005112, Databank URL http://ngdc.cncb.ac.cn/gsa.
