# Supplementary material for: A missense mutation in Pitx2 leads to early-onset glaucoma via NRF2-YAP1 axis
Source: Cell Death Dis. 2021 Oct 29;12(11):1017. doi: 10.1038/s41419-021-04331-1 (PMC8556256; doi:10.1038/s41419-021-04331-1)
Supplement: Supplementary file 1 — Supplemental data [file 41419_2021_4331_MOESM1_ESM.pdf]

A missense mutation in *Pitx2* leads to early onset glaucoma via NRF2-YAP1 axis

Yeming Yang[1,2,6#], Xiao Li[1#], Jieping Wang[3], Junkai Tan[4], Bernie Fitzmaurice[3], Patsy M. Nishina[3], Kuanxiang Sun[1], Wanli Tian[1], Wenjing Liu[1], Xuyang Liu[4,5*], Bo Chang[3]*, Xianjun Zhu[1,2,6]*

Supplemental data include Figures S1-12 and Tables S1-4.

Supplemental Figures

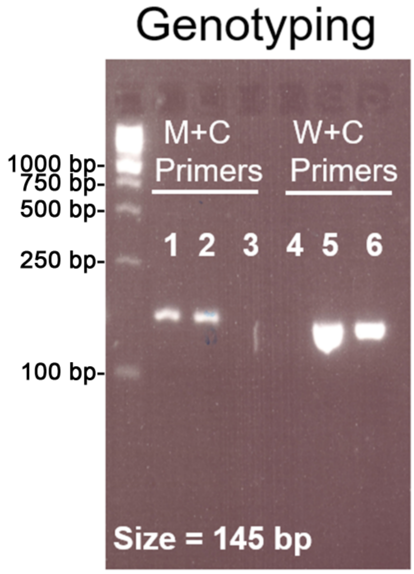

Figure S1. Allele-specific PCR on genomic DNA confirmed the presence of the *Pitx2*^{egl1} mutation.

Three oligo primers were selected from exon 2 of *Pitx2* gene with [G/T] single base change (WT=G, *egl1* mutant=T), namely wild-type (W), mutant (M) and common (C) primers. The AS-PCR assay was conducted in two parallel experiments: one using “mutant + common” primers (M+C) and the other using “wild + common” primers (W+C). Lanes 1 and 4 were homozygous mutants (*egl1/egl1*), Lanes 2 and 5 were heterozygous mutants (*egl1/+*), and Lanes 3 and 6 were wild type (+/+). The mutant band appeared in homozygous (1) and heterozygous mutant (2), and no band in wild

type (3) in M+C primer experiment; the wild type band appeared in wild type (6) and heterozygous mutant (5), and with no band in homozygous mutant (4) in W+C primer experiment.

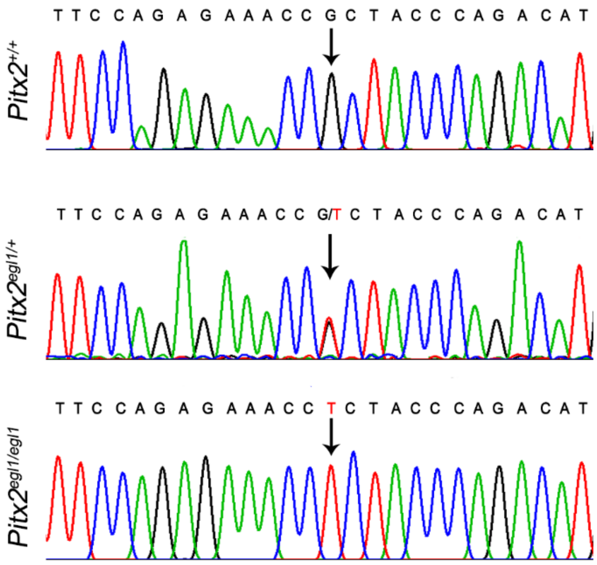

Figure S2. Sanger sequencing confirmed the *Pitx2^{egl1}* mutation.

Representative Sanger sequencing results of the WT (+/+), heterozygous mutants (*egl1/*+) and homozygous mutants (*egl1/egl1*). Note a transversion substitution c.G344T in *egl1* mutant.

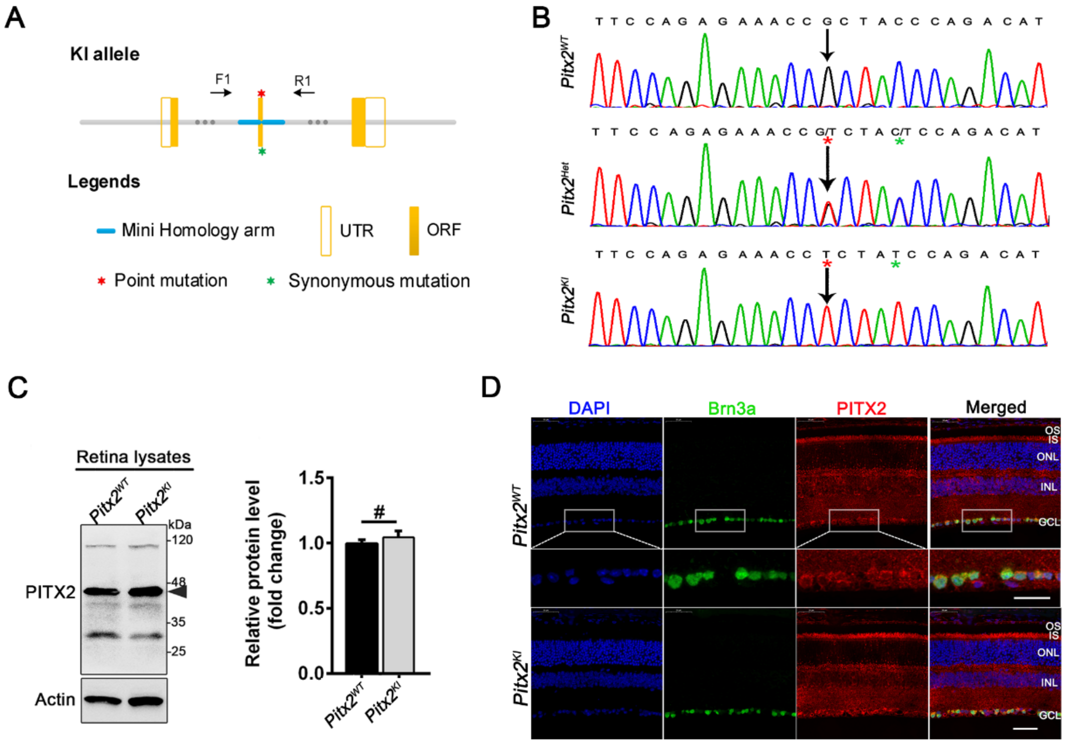

Figure S3. Generation of $Pitx2^{KI}$ mutant mice and analysis of the *Pitx2* mutation.

(A) Targeting strategy of $Pitx2^{KI}$ mutant mice construction using CRISPR/Cas9 oligonucleotide directed repair technique.

(B) Representative Sanger sequence results of the *Pitx2* WT ($Pitx2^{WT}$), heterozygote ($Pitx2^{Het}$) and mutant ($Pitx2^{KI}$) mice used in this study.

(C) Immunoblotting of PITX2 protein expression in retinas from $Pitx2^{WT}$ or $Pitx2^{KI}$ mice. β-Actin was used as a loading control. WT and mutant PITX2 protein expressed at similar levels in retina.

(D) Immunofluorescence labelling of retinal cryosections from $Pitx2^{WT}$ and $Pitx2^{KI}$ mice at age of 2 months using a mouse anti-PITX2 antibody. The Brn3a antibody was used to mark ganglion cells. DAPI was used to counterstain the nuclei. OS, outer segment; IS, inner segment; ONL, outer nuclear layer; INL, inner nuclear layer; GCL, ganglion cell layer. Scale bar, 20 µm. #, not significant. Data are presented as the mean ± SEM.

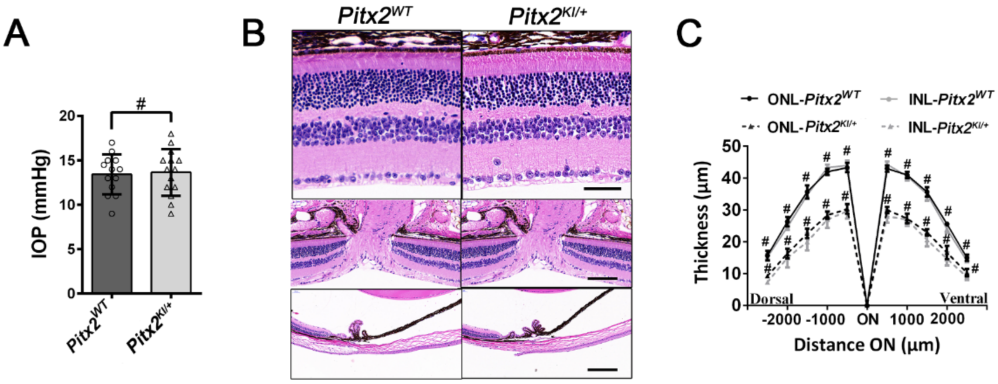

Figure S4. Heterozygous *Pitx2$^{KI/+}$* mice showed normal IOP and retinal structure. (A) Intraocular pressure analysis in the *Pitx2^{WT}* and *Pitx2$^{KI/+}$* mice at 3 months of age (n = 12). Individual IOP values were shown by dots in the histograms.

(B) Representative H&E-stained sections of 3-month-old *Pitx2^{WT}* and *Pitx2$^{KI/+}$* mice retinas. The upper panels show histological sections of retinas, Scale bars: 25 μm; The middle panels show images of optic nerve heads, Scale bars: 100 μm; The bottom panels show images of the anterior chamber. Scale bars: 50 μm.

(C) Statistical analysis of ONL and INL thickness (n = 4). #, no significant difference. The data are represented as mean ± SEM.

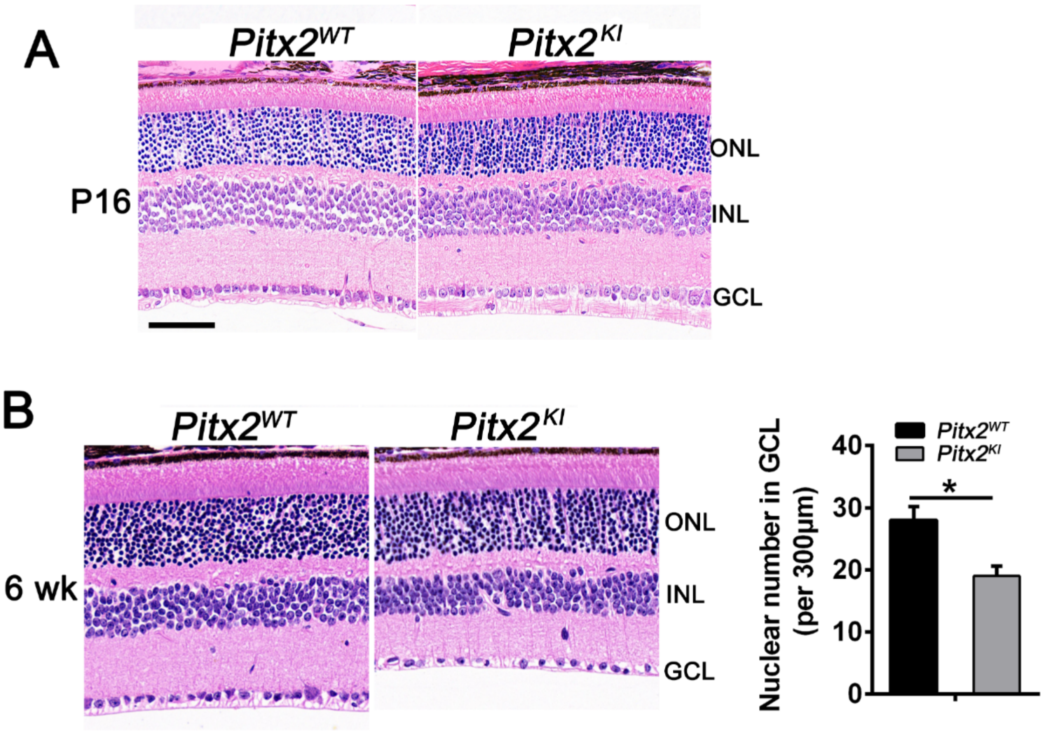

Figure S5. Histological examination of retinas from P16 and 6-week-old WT and mutant mice.

(A) H&E-stained sections of P16 *Pitx2*^{WT} and *Pitx2*^{KI} retinas. Scale bars: 25 μm

(B) H&E-stained sections of 6-week-old *Pitx2*^{WT} and *Pitx2*^{KI} retinas. Scale bars: 25 μm

(C) Quantification of the nuclei number in the GCL of 6-week-old mice retinas. The *Ptix2* mutants presented a decreased nuclei number in the GCL (n = 4). *p < 0.05. The data are presented as mean ± SEM.

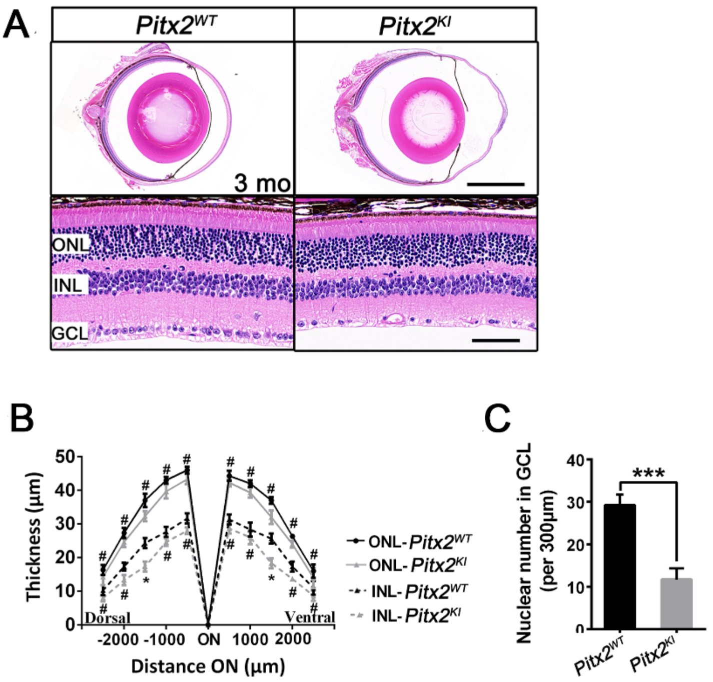

Figure S6. Histological examination of retinas from 3-month-old WT and mutant mice.

(A) H&E-stained sections from 3-month-old WT and *Pitx2*^{KI} mutant retina. The upper panels show the entire retina. Scale bars: 1000 μm. The bottom panels are higher-magnification images of the neural retina. Scale bars: 50 μm.

(B) *Pitx2*^{KI} mutant mice at 3 months of age exhibit slightly thinner inner and outer nuclear layers compared with WT mice (n=6 in each group).

(C) Quantification of the nuclei number in GCL (n=6 in each group). The *Pitx2*^{KI} mutant mice have a significantly decreased number of nuclei in their GCL. *$p < 0.05$; ***$p < 0.001$. #, no significant difference. The data are presented as mean ± SEM.

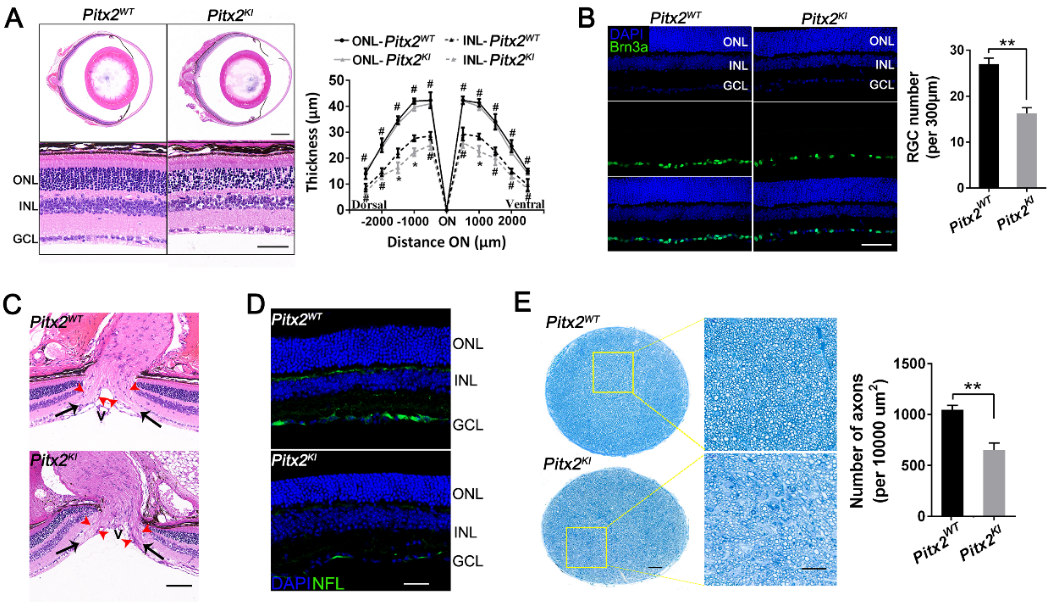

Figure S7. The pathological changes in mutants with normal IOPs.

(A) H&E-stained retinal sections from 3-month-old WT and *Pitx2*^{KI} mice with normal IOP. The upper panels show the entire retina. Scale bars: 1000 μm. The bottom panels are higher-magnification images of the neural retina. Scale bars: 50 μm. Right panel shows the quantification of the nuclei number in GCL (n=6 for each group).

(B) Immunofluorescent image of retinal cryosections from WT and mutants with normal IOP following labelling with anti-Brn3a to label RGCs. Scale bars: 50 μm. *Pitx2*^{KI} mutants have significantly decreased number of RGCs per mm^2 relative to their control littermates (n=6 for each group).

(C) Representative images of histologic sections taken through the ON heads from *Pitx2*^{WT} and *Pitx2*^{KI} mice with normal IOP. The ON head appeared abnormal in *Pitx2*^{KI} mice with normal IOP, with thinning of the NFL (red arrowheads) and optic nerve excavation (asterisk). Black arrows indicate NFL entering the ON; V: Central blood vessel. Scale bars: 50 μm.

(D) Immunofluorescent staining of retinal cross sections were shown. Retinal nerve fibers were labelled with neurofilament-L (NF-L), and the nuclei were counter-stained with DAPI. Scale bars: 25 μm.

(E) ON atrophy was assessed in cross semi-thin sections of resin-embedded optic nerves from 3-month-old WT and mutant mice with normal IOP stained with toluidine

blue staining. Scale bars: 100 μm. The panels on the right show high-magnification images of the boxed areas shown in the panels on the left. Scale bars: 10 μm. The *Pitx2*^{KI} mutants with normal IOP have significantly less axonal projections compared to *Pitx2*^{WT} mice (n=12 in each group). ONL, outer nuclear layer; INL, inner nuclear layer; GCL, ganglion cell layer. *, P<0.05; **p < 0.01; ***p < 0.001. #, no significant difference. The data are presented as mean ± SEM.

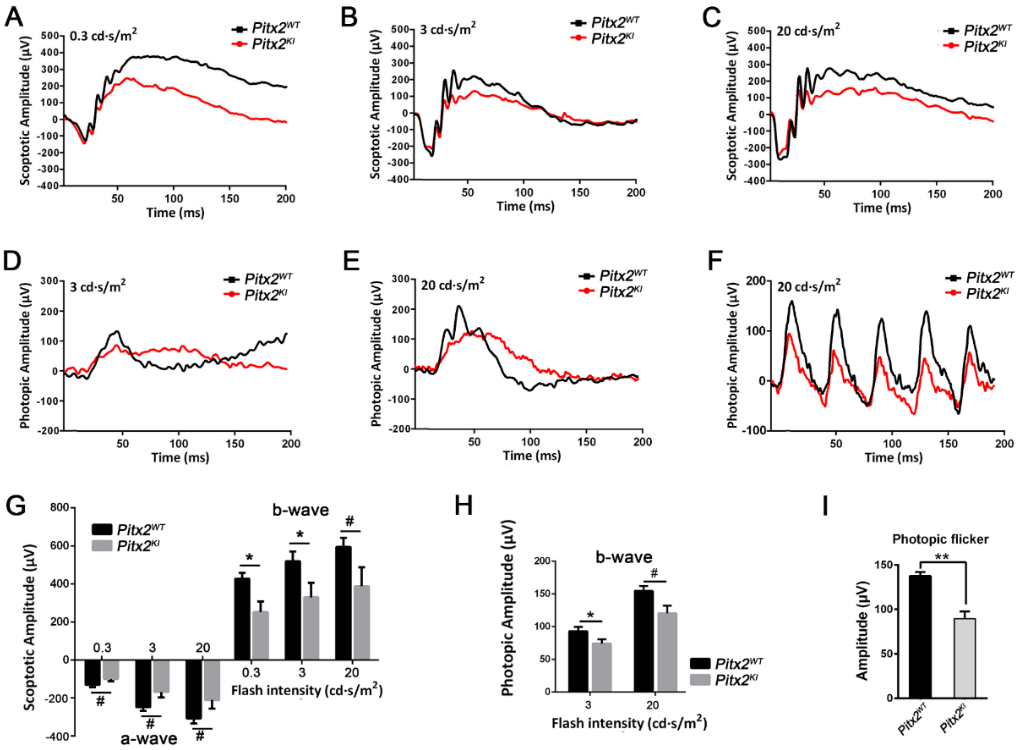

Figure S8. Impaired visual function of *Pitx2* mutant mice

(A-C) Representative electroretinogram (ERG) traces corresponding to responses elicited by scotopic conditions at flash intensities from 0.3, 3, and 20 cd sec/m^2 in mice at 3 months of age.

(D-F) Representative ERG traces corresponding to responses elicited by photopic conditions at flash intensities of 3 and 20 cd sec/m^2 in mice at 3 months of age.

(G-H) Statistical analysis of amplitudes of scotopic a- and b-waves (G) and photopic b-wave (H) electroretinograms.

(I) T-test was performed for photopic flicker of mice at 3 months. The sample size was n=4 for both the control and mutant groups. *p < 0.05; **p < 0.01; #, not significant.

Data are presented as the mean ± SEM.

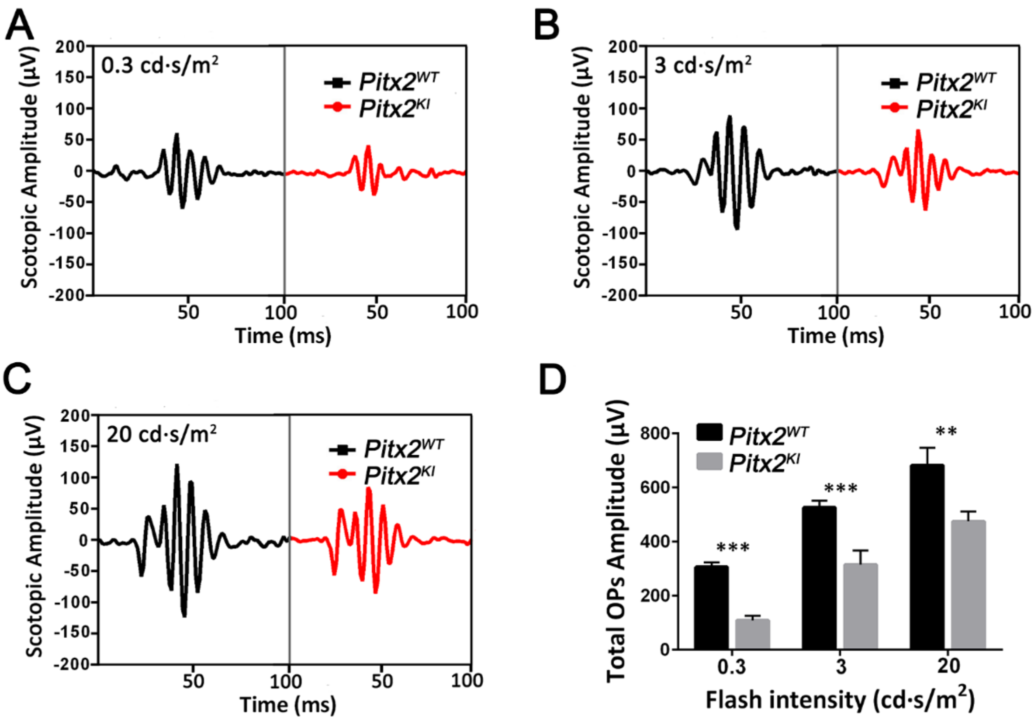

Figure S9. ERG test revealed decreased total OPs amplitude of *Pitx2* mutant mice.

(A-C) Waveforms of OPs under scotopic conditions at flash intensities of 0.3, 3 and 20 under scotopic conditions at flash intensities from 0.3, 3, and 20 cd sec/m^2 in mice at 3 months of age.

(D) Statistical analysis of the amplitudes of OPs in the mice under scotopic conditions are presented. The sample size was n = 4 for both the WT and mutant groups. **p < 0.01; ***p < 0.001. The data are represented as mean ± SEM.

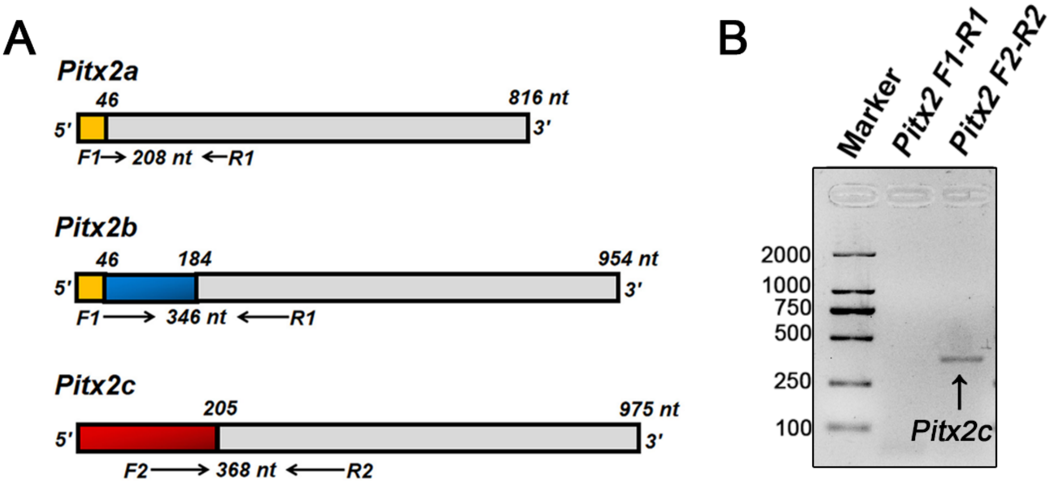

Figure S10. Identification of *Pitx2* isoform expression in mouse retina.

(A) Schematic representation of three *Pitx2* cDNA isoforms (*Pitx2a-c*) in mouse.

(B) Only the *Pitx2c* isoform was detected in the cDNA samples from WT mouse retina by RT-PCR.

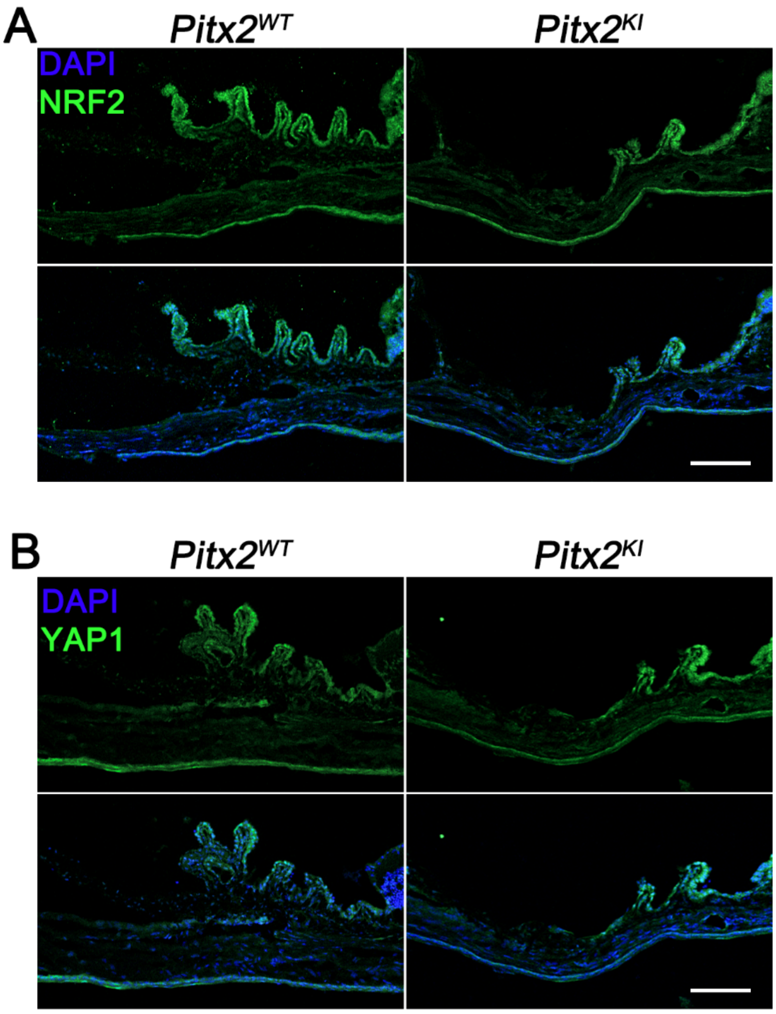

Figure S11. The expression levels of NRF2 and YAP1 were not changed in the outflow tissues of mutants.

(A-B) Ocular frozen sections from 7-week-old WT and mutants were immunolabelled using antibodies against NRF2 (A, green) and YAP1 (B, green). The nuclei were counter-stained with DAPI (blue). Scale bars: 50 μm.

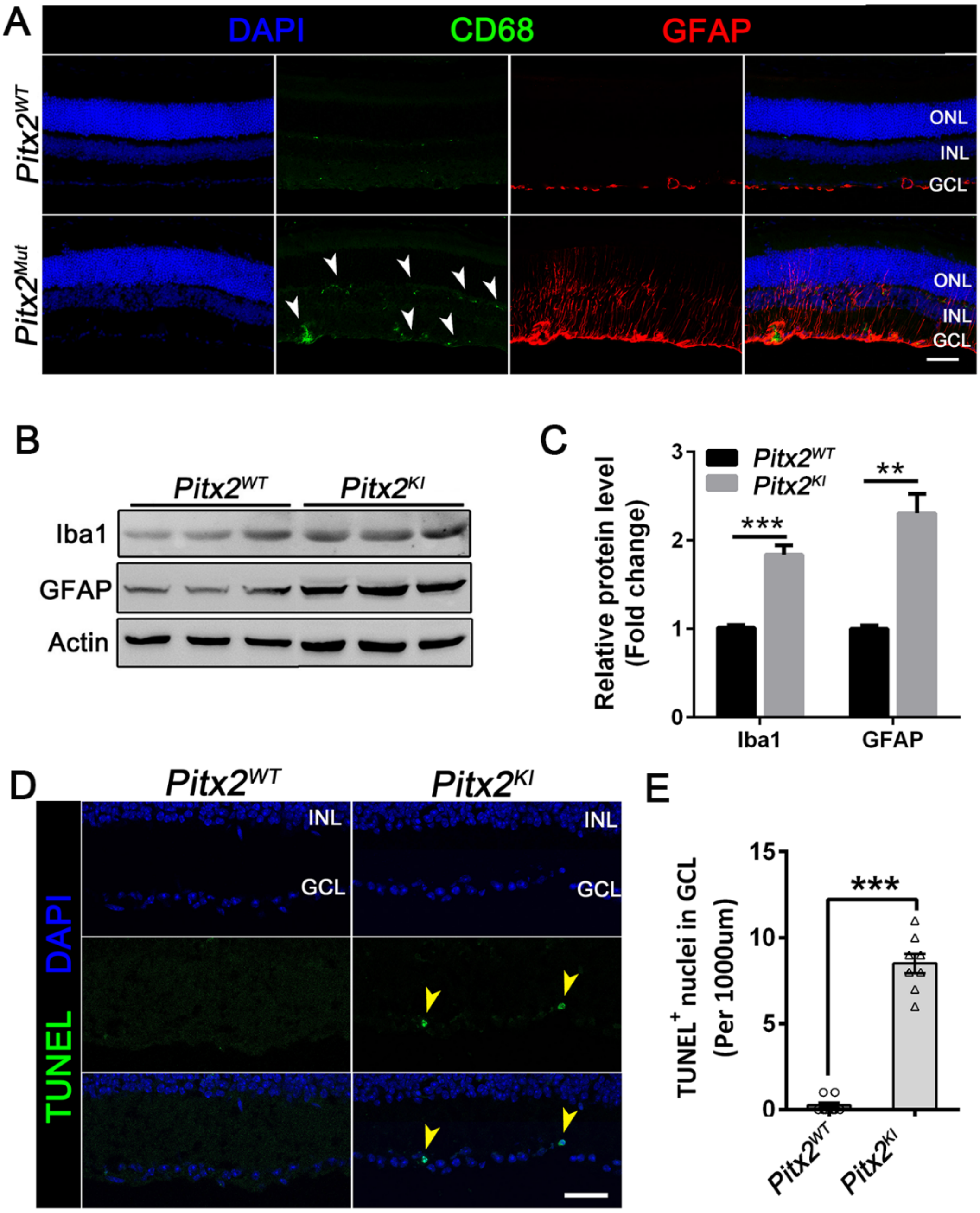

Figure S12. Inflammatory response and apoptosis in *Pitx2^{KI}* retinas.

(A) Cryosections from the retinas of 2-month-old *Pitx2^{WT}* and *Pitx2^{KI}* mice were co-immunostained for the activated microglial marker CD68 (green) and the activated astrocyte marker GFAP (red). Nuclei were counter-stained with DAPI (blue). Arrows indicate activated microglia with increased CD68 expression. Sample size n=4 for both *Pitx2^{WT}* and *Pitx2^{KI}* retinas. Scale bar: 50 μm.

(B) Representative immunoblots of 2-month-old retina lysates from *Pitx2^{WT}* and *Pitx2^{KI}* mice reacted with antibodies to Iba1 and GFAP. β-actin was used as the

loading control. Uncropped immunoblotting images were shown in Figure S9.

(C) Quantification of immunoblots of retina lysates from *Pitx2*^{WT} and *Pitx2*^{KI} mice. Both Iba1 and GFAP levels (normalized to control) in *Pitx2*^{KI} retinas were elevated compared with levels in *Pitx2*^{WT} retinas.

(D) Immunofluorescence labelling of retina cryosections from 2-month-old *Pitx2*^{WT} and *Pitx2*^{KI} littermates with the TUNEL assay kit. Nuclei were counter-stained with DAPI (blue). TUNEL-positive cells (green) were observed in GCL of the *Pitx2*^{KI} retina sections. Arrows represent TUNEL-positive cells. Sample size n=6 for both *Pitx2*^{WT} and *Pitx2*^{KI} retinas. Scale bar: 25 µm.

(E) Quantitative assessment of the number of TUNEL-positive cells in GCL of whole section. Sample size n=6 for both *Pitx2*^{WT} and *Pitx2*^{KI} retinas. ONL, outer nuclear layer; INL, inner nuclear layer; GCL, ganglion cell layer. $^{**}p < 0.01$, $^{***}p < 0.001$. The data are represented as means ± SEM.

Table S1. Filtered candidate genes of WES (See the attachment).

Table S2. Variants between D3Mit106 and D3Mit291 on Chromosome 3 (See the attachment).

Table S3. Immunological antibodies used in this study

Antibody	Dilution rate for IHC	Dilution rate for WB	Species	Company	Cat. No.
Anti-PITX2	1:200	1:1000	Mouse	Novus	H00005308-M01
Anti-CD68	1:100	_	Rat	Abcam	ab53444
Anti-GFAP	1:200	1:2000	Rabbit	CST	80788S
Anti-Iba1	_	1:1000	Rabbit	Abcam	ab178846
Anti-Brn3a	1:200	_	Rabbit	Abcam	ab245230
Anti-NF-L	1:200	_	Rabbit	Abcam	ab223343
Anti-Flag	1:200	1:2000	Mouse	Sigma	ab169276
Anti-β-actin	_	1:2000	Rabbit	CST	4967
Anti-HA	_	1:5000	Rat	Roche	11867423001
Anti-Calnexin	1:200	_	Rabbit	Abcam	ab22595
Anti-NRF2	1:200	1:1000	Rabbit	Abcam	ab31163
Anti-αSMA	1:200	_	Rabbit	Abcam	ab124964

Table S4. Primers used in genotype and qPCR

Number	Primer	Sequence (5' to 3')
1	Pitx2-G344T-F	CAGGTGCCGAGCACAGACCTTTC
	Pitx2-G344T-R	TGGGAACCGGGAGCAGTAACCT
2	Pitx2-cDNA-F1	GCAGCCGTTGAATGTCTCTT
	Pitx2-cDNA-R1	TCTTGAACCAAACCCGGACT
3	Pitx2-cDNA-F2	ATCTCCGATACTTCCAGCCC
	Pitx2-cDNA-R2	TAGCCGGGGTACATGTCATC
4	Cyp2a5-F	GAGATTTCCTCCTCCCCAAG
	Cyp2a5-R	TAGCCAGTCCTTCTCCGAAA
5	Upk3bl-F	CCCACGGAAGGTAGAAGACA
	Upk3bl-R	GAGCTTCTCGGAATGTCTGG
6	Rrm2-F	GATCGTGTGTTCTTCGCTGA
	Rrm2-R	TTGCTTGATGGTCACTGCTC
7	Cyp26a1-F	TTCGGGTTGCTCTGAAGACT
	Cyp26a1-R	TCCTCCAAATGGAATGAAGC
8	Plet1-F	CTTCCACACCTGGGACTGTT
	Plet1-R	TCACTGCTTTCAGGATCACG
9	Slc25a24-F	CGGCTAGCTGTAGCCAAAAC
	Slc25a24-R	GATAGGGCTCCACAGCTCAG
10	Cyp2s1-F	CTGCTCCTGCTGAGATACCC
	Cyp2s1-R	CAGAGGGAAGACCTCAGTGC
11	Cyp2f2-F	TGCAGGAAGAGATTGACCGT
	Cyp2f2-R	TGTGATGACATCTGTGCCCT
12	Cyp26b1-F	CTGCATCTCTGCCAGGTGTA
	Cyp26b1-R	AAAGGGGCAGAGAGCTAAGG
13	Cyp4a12b-F	TCATGAAGTGTGCCTTCAGC
	Cyp4a12b-R	TGTGTGTCATGGGCAAGTT
