# Supplementary material for: A missense mutation in Pitx2 leads to early-onset glaucoma via NRF2-YAP1 axis
Source: Cell Death Dis. 2021 Oct 29;12(11):1017. doi: 10.1038/s41419-021-04331-1 (PMC8556256; doi:10.1038/s41419-021-04331-1)

Full unedited gel for Figure 5A

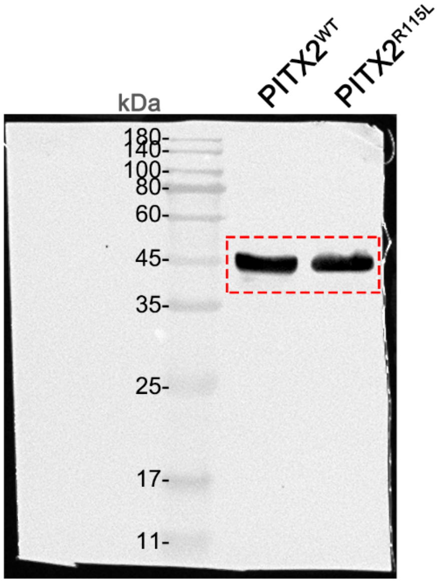

IB: Flag

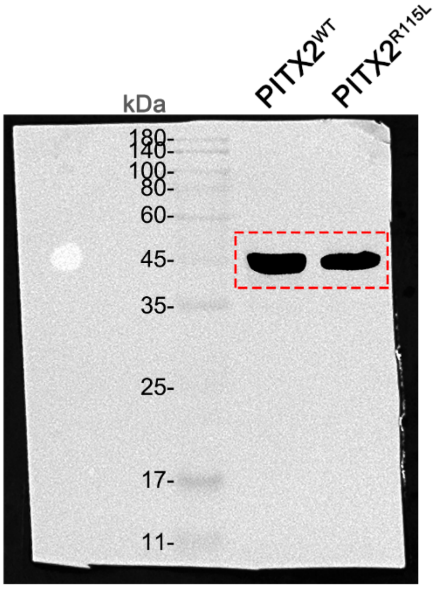

IB: Actin

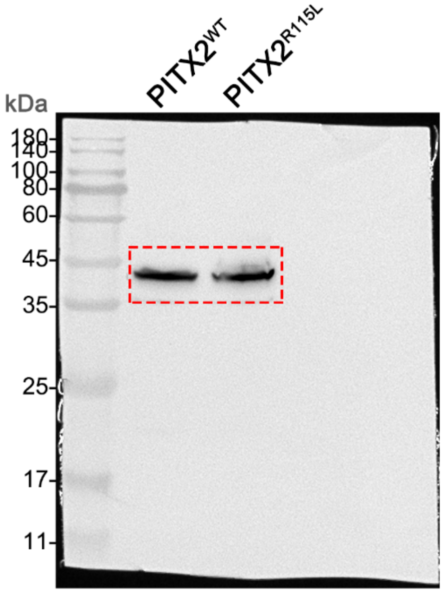

IB: PITX2

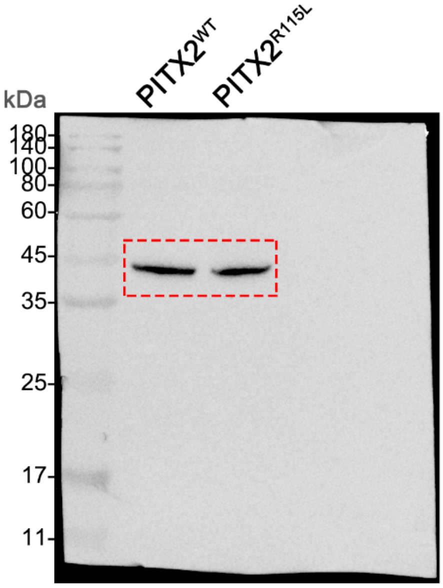

IB: Actin

Full unedited gel for Figure 5D,E

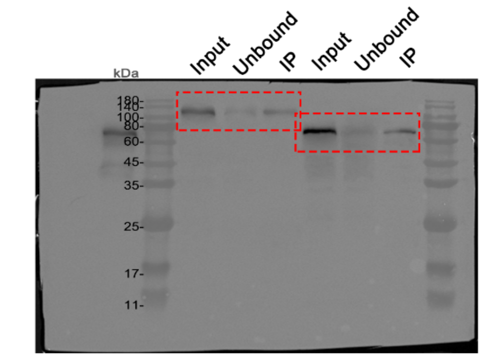

IB: HA (NRF2/YAP1)

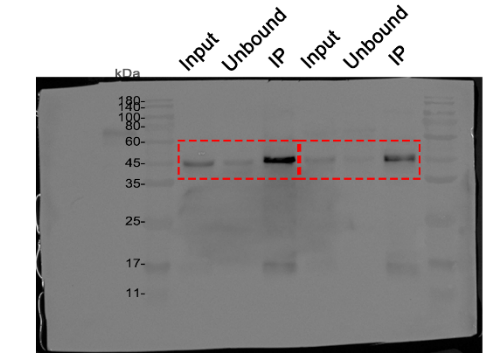

IB: Flag (PITX2)

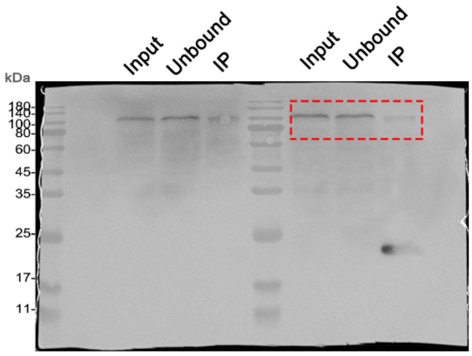

IB: HA (NRF2)

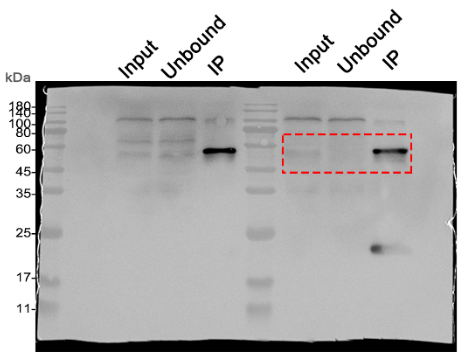

IB: Flag (PITX2)

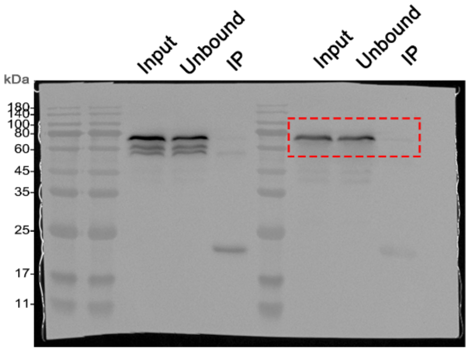

IB: HA (YAP1)

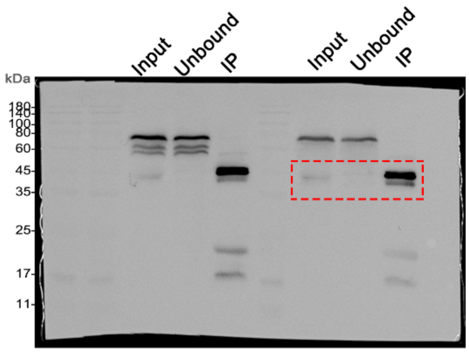

IB: Flag (PITX2)

Full unedited gel for Figure 6G, Figure S12B

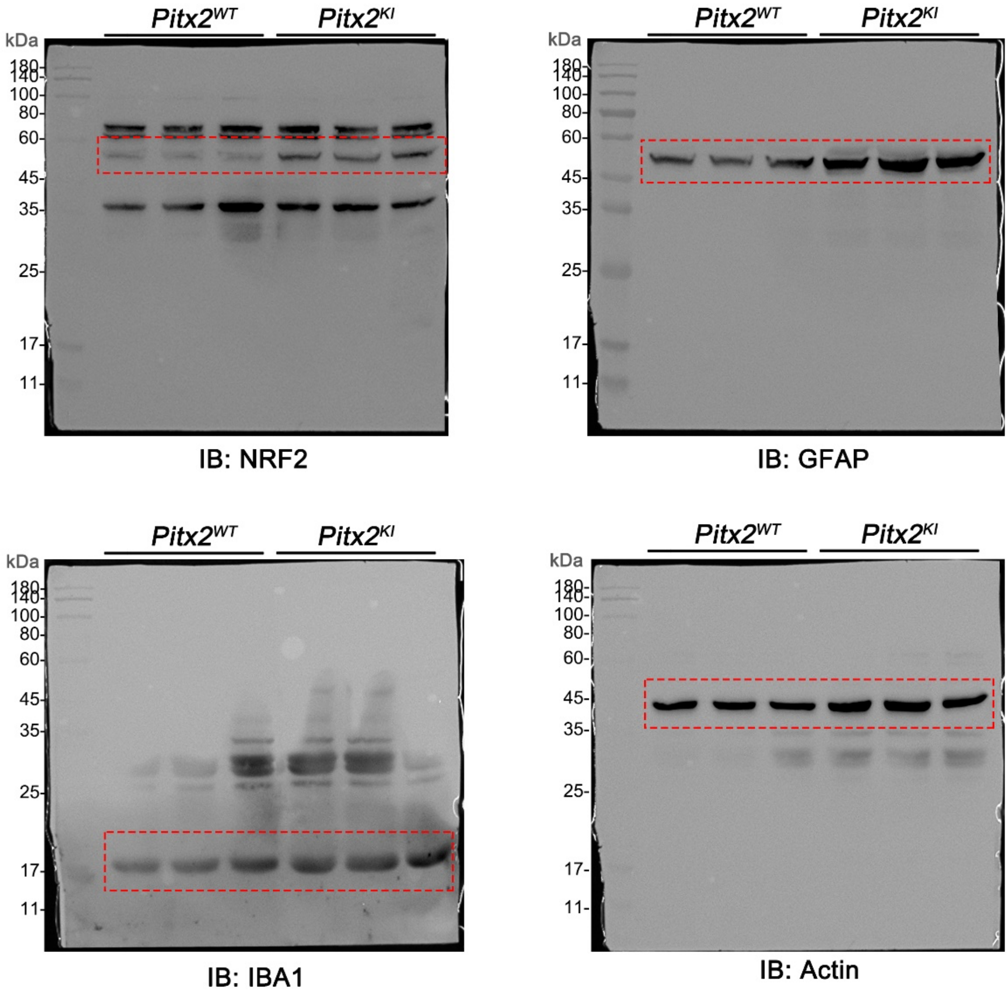

Supplement: Supplementary file 4 — Uncropped gel images [file 41419_2021_4331_MOESM4_ESM.pdf]
